# Sono‐Controllable Janus Hydrogel Platform for Sequential Tumor Eradication and Bone Regeneration in Metastatic Breast Cancer

**DOI:** 10.1002/advs.202506386

**Published:** 2025-09-04

**Authors:** Yitong Li, Yunyun Liu, Chuyun Lou, Xuejun Chen, Ying Zhang, Hui Shi, Jiayi Qiu, Shen Zhang, Taixia Wang, Xiaowei Wang, Haohao Yin, Huixiong Xu, Yifeng Zhang

**Affiliations:** ^1^ Department of Ultrasound Shanghai Tenth People's Hospital Tongji University School of Medicine Shanghai 200072 P. R. China; ^2^ Department of Ultrasound Institute of Ultrasound in Medicine and Engineering Zhongshan Hospital Fudan University Shanghai 200032 P. R. China; ^3^ Shanghai Engineering Research Center of Ultrasound Diagnosis and Treatment National Clinical Research Center for Interventional Medicine Shanghai 200072 P. R. China; ^4^ Department of Radiology The First Affiliated Hospital of Zhengzhou University Zhengzhou University Zhengzhou Henan Province 450052 P. R. China; ^5^ Department of Ultrasound Zhongshan Hospital (Xiamen) Fudan University Fujian Province Xiamen 361015 P. R. China; ^6^ Department of Stomatology Shanghai Tenth People's Hospital Tongji University Cancer Center School of Medicine Tongji University Shanghai 200072 P. R. China; ^7^ Department of Ultrasound Shanghai General Hospital Shanghai Jiao Tong University School of Medicine Shanghai 200080 P. R. China

**Keywords:** bone metastases, bone regeneration, Janus hydrogel, necroptosis, reactive oxygen species, tumor eradication

## Abstract

Bone metastases occur in 60%–75% of patients with metastatic breast cancer, reducing survival rates and compromising quality of life. Innovative treatments are urgently needed to sequentially eradicate tumor cells and promote bone regeneration. In this study, a novel Janus hydrogel platform (GA@CaMP) is developed for encapsulating the sonosensitive composite nanomaterial MHP, which enables gene expression regulation, along with oxygen‐releasing CaO_2_ NPs. The platform's therapeutic efficacy is achieved through two distinct mechanisms: high‐concentration ROS, activated under specific ultrasonic conditions, synergizes with the upregulation of ZBP1 expression to co‐activate the necroptotic pathway, inducing tumor cell death and effectively eliminating residual cancer cells. Subsequently, low‐concentration ROS stimulates osteogenic gene expression, promoting bone regeneration in affected areas. The incorporation of CaO_2_ NPs further enhances therapeutic outcomes through continuous oxygen release, which improves the local hypoxic microenvironment and consequently promotes more effective tumor eradication and bone regeneration. This multifunctional Janus hydrogel system demonstrates remarkable efficacy in tumor clearance and bone defect repair, representing a significant advancement in the comprehensive treatment of bone metastatic breast cancer. The platform's dual functionality and therapeutic precision position it as a promising strategy for holistic breast cancer management, with substantial potential for clinical translation and application in cancer therapy.

## Graphical abstract

Sequential tumor eradication and bone regeneration remain a significant challenge for patients with breast cancer bone metastases. This study develops a novel Janus hydrogel platform, which can achieve tumor elimination and bone repair under the action of different ultrasound frequencies. Moreover, the sustained‐release oxygen can improve the hypoxic microenvironment for synergistic enhancement of efficacy.

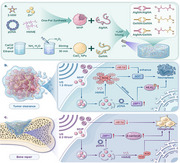

## 1 Introduction

Breast cancer bone metastasis is one of the most common complications of breast cancer, affecting approximately 65%–75% of patients with metastatic breast cancer.^[^ ^1^ ^]^ According to the SEER database, the 3‐year survival rate for patients with breast cancer bone metastasis is only 50.5%, with a median survival time of just 36 months.^[^ ^2^ ^]^ In addition to significantly increasing mortality, these patients often experience bone pain, pathological fractures, hypercalcemia, and other bone‐related events, which severely impair their quality of life. Currently, the treatment of breast cancer bone metastasis primarily focuses on alleviating symptoms and delaying disease progression.^[^ ^3^ ^]^ However, surgery and radiotherapy are limited by their inability to achieve complete tumor eradication, while chemotherapy and targeted therapies are hindered by the bone–blood barrier, which restricts effective drug delivery and often causes severe side effects, resulting in suboptimal treatment outcomes.^[^ ^3^, ^4^ ^]^ Additionally, as breast cancer bone metastases are frequently osteolytic, reconstructing bone defects caused by tumor invasion presents significant challenges.^[^ ^4^, ^5^ ^]^ Traditional bone repair methods, such as bone grafts, can restore bone structure and function to some extent but are limited by donor material shortages and the risk of immune rejection after transplantation.^[^ ^6^, ^7^ ^]^ Furthermore, the repair of bone defects relies not only on material filling but also on promoting new bone formation and regulating the microenvironment during the healing process. As breast cancer bone metastases are typically associated with alterations in the tumor microenvironment, such as increased acidity and hypoxia, conventional bone repair strategies often fail to address these specific challenges, resulting in limited repair outcomes.^[^ ^8^ ^]^ Moreover, cancer cell re‐invasion must be avoided during the repair process to minimize the risk of recurrence. Therefore, a dual‐function strategy that combines anti‐tumor effects with bone repair promotion is of significant clinical value.

In recent years, reactive oxygen species (ROS) have garnered attention as important intracellular signaling molecules, demonstrating unique bidirectional regulatory roles in tumor therapy and tissue repair. High concentrations of ROS can induce apoptosis and necrosis in tumor cells by oxidatively damaging cellular DNA, lipids, and proteins.^[^ ^9^, ^10^ ^]^ However, excessive ROS not only harms tumor cells but also disrupts surrounding normal tissues and cells, which limits their clinical application.^[^ ^11^ ^]^ In contrast, low concentrations of ROS promote osteogenesis. Research has shown that low levels of ROS can stimulate the proliferation, differentiation, and mineralization of bone marrow‐derived mesenchymal stem cells (BMSCs) by modulating signaling pathways such as the mitogen‐activated protein kinase (MAPK) pathway, the Wnt/β‐catenin pathway, and the nuclear factor erythroid 2‐related factor 2 (Nrf2) pathway, thereby accelerating bone repair.^[^ ^12^, ^13^, ^14^ ^]^ Additionally, ROS can regulate cellular membrane oxidation, activate antioxidant responses, and enhance repair mechanisms, facilitating bone tissue regeneration.^[^ ^15^ ^]^ This bidirectional regulatory effect makes ROS an ideal therapeutic molecule, but achieving precise control over ROS levels, particularly to meet the dual demands of tumor treatment and bone repair, remains a significant challenge.

The controllability and high targeting ability of ultrasound (US) enable the spatially and temporally controlled release of ROS on demand.^[^ ^12^, ^16^ ^]^ By adjusting the US frequency, ROS release can be precisely regulated to meet specific treatment needs. Additionally, as a non‐invasive treatment modality, US possesses remarkable deep tissue penetration, making it an ideal choice for treating deep‐seated tumor tissues.^[^ ^17^ ^]^ However, although regulating ROS concentrations can exert both anti‐tumor and bone repair‐promoting effects to some extent, its efficacy remains limited in complex pathological conditions such as breast cancer bone metastasis. The hypoxic tumor microenvironment is one of the key factors affecting ROS treatment efficacy. Under hypoxic conditions, the expression of HIF‐1α is upregulated, enabling tumor cells to enhance metabolic pathways and antioxidant capabilities to adapt to high ROS concentrations, thereby reducing ROS‐induced cytotoxicity.^[^ ^18^ ^]^ The weakened mitochondrial function and limited ROS production further decrease ROS effectiveness in tumor therapy.^[^ ^19^ ^]^ Moreover, hypoxia also activates immune‐suppressive signals, which modulate immune cell functions, promoting immune escape and immune suppression.^[^ ^20^ ^]^ Simultaneously, the hypoxic microenvironment significantly affects the bone repair process. Under low‐oxygen conditions, the proliferation, migration, and differentiation of osteoblast precursor cells are inhibited, delaying new bone formation. Hypoxia also reduces the osteogenic differentiation capacity of BMSCs, prolonging functional recovery and suppressing local immune functions, thereby diminishing the efficiency of bone repair.^[^ ^21^, ^22^ ^]^ Therefore, oxygen supplementation plays a critical role in both tumor therapy and bone repair.

Changes in gene expression, particularly the dysregulation of key genes, can also impact the efficacy of ROS‐based therapy. ZBP1 (Z‐NA binding protein 1), an important cytosolic nucleic acid sensor and a key downstream effector of the cGAS‐STING pathway, has shown promise in tumor therapy.^[^ ^23^, ^24^, ^25^ ^]^ ZBP1 activates necroptotic pathways and induces cytokine release, enhancing cellular responses to ROS and promoting tumor cell death. Studies indicate that ZBP1 expression is typically low in tumors characterized by genomic instability, such as triple‐negative breast cancer, which leads to enhanced oxidative stress tolerance and inadequate immune response, further complicating treatment.^[^ ^25^ ^]^ Upregulating ZBP1 expression can effectively increase tumor cell sensitivity to ROS, activate its downstream cell death pathways, and thereby improving therapeutic efficacy. Notably, ZBP1 also plays a significant role in bone repair. Research shows that ZBP1 regulates the Wnt/β‐catenin signaling pathway, promoting osteogenic differentiation of BMSCs and accelerating bone repair.^[^ ^26^ ^]^ Thus, modulating ZBP1 expression may not only enhance ROS‐mediated tumor cell killing but also promote bone repair, offering dual therapeutic benefits. Therefore, a multidimensional therapeutic strategy integrating the on‐demand regulation of ROS, ZBP1 gene modulation, and alleviation of the hypoxic microenvironment may be the key to achieving both anti‐tumor and bone repair effects.

**Figure 1:**
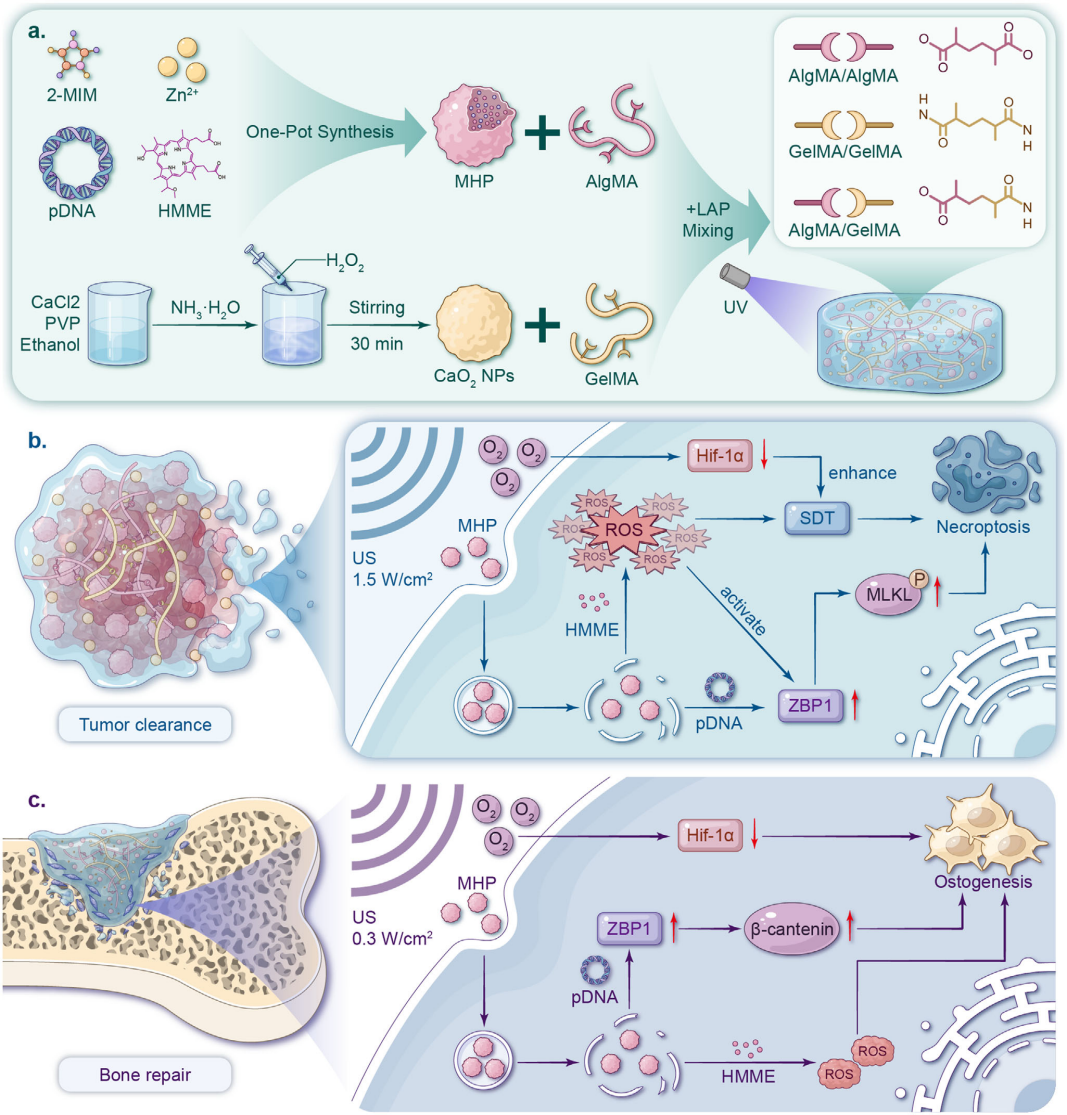
Schematic illustration of the injectable GA@CaMP hydrogel platform for effective treatment of breast cancer bone metastases and simultaneous bone tissue repair under US irradiation at different frequencies. a) Preparation of the GA@CaMP hydrogel platform. b) Schematic showing the mechanism by which the in situ injectable GA@CaMP, containing MHP and CaO_2_ NPs, eliminates tumors. Under high‐frequency US irradiation, MHP generates abundant ROS to induce sonodynamic tumor cell destruction. Synergized by the ZBP1 plasmid, they jointly activate the MLKL pathway to promote necroptosis in tumor cells. Additionally, O_2_ released from CaO_2_ significantly downregulates HIF‐1α, further enhancing the sonodynamic therapeutic effect. c) Schematic illustrating the mechanism by which GA@CaMP induces osteogenic differentiation in bone defects. Under low‐frequency US irradiation, a moderate amount of ROS produced by MHP, together with the ZBP1 plasmid, promotes the osteogenic differentiation of bone marrow mesenchymal stem cells (BMSCs). Additionally, O_2_ released from CaO_2_ significantly downregulates HIF‐1α, creating a favorable environment for osteogenic proliferation and differentiation during subsequent bone repair.

Herein, we have designed and constructed a multifunctional Janus hydrogel system, GA@CaMP. This system combines sonosensitive composite nanoparticles (MHP), calcium peroxide nanoparticles (CaO_2_ NPs), and a gelatin methacryloyl/alginate methacrylate hybridized hydrogel (GelMA/AlgMA) to precisely control ROS release via US (**Figure** **1**). Specifically, MHP is composed of the ZBP1 plasmid and sonosensitizer HMME encapsulated within a metal–organic framework (ZIF‐8). The highly ordered, stable ZIF‐8 structure ensures the stability and bioactivity of the encapsulated materials, and its unique acid‐responsive release mechanism ensures that therapeutic agents are released only in the diseased area, minimizing toxicity and side effects to healthy tissues.^[^ ^27^, ^28^ ^]^ MHP nanoparticles serve both as sonosensitive nanomaterials that release ROS under US stimulation and as a gene delivery platform for precise regulation of ZBP1 expression. The CaO_2_ NPs act as oxygen generators, producing oxygen at tumor and bone defect sites to alleviate hypoxia, enhance tumor treatment, and promote osteogenic differentiation of BMSCs, thereby supporting both tumor therapy and bone regeneration. GelMA/AlgMA composite hydrogels serve as carriers, providing an ideal biocompatible platform. The combination of GelMA and AlgMA mimics the extracellular matrix, providing excellent biocompatibility and a porous structure that facilitates nutrient delivery while supporting BMSCs adhesion and growth. More importantly, the sustained release properties of GelMA/AlgMA ensure the long‐term stability of functional components, preventing rapid metabolism or elimination and thereby enhancing the persistence and efficacy of treatment. Through the synergistic action of these components, our Janus hydrogel system achieves dual effects in both tumor treatment and bone repair. This innovative integrated sequential treatment strategy provides a promising approach for breast cancer bone metastasis therapy, demonstrating the vast potential of multidimensional therapeutic strategies and offering new insights and technical pathways for future cancer treatment and tissue repair.

## 2 Results and Discussion

### 2.1 Preparation, Characterization, and Performance of MHP and CaO_2_ NPs

**Figure 2:**
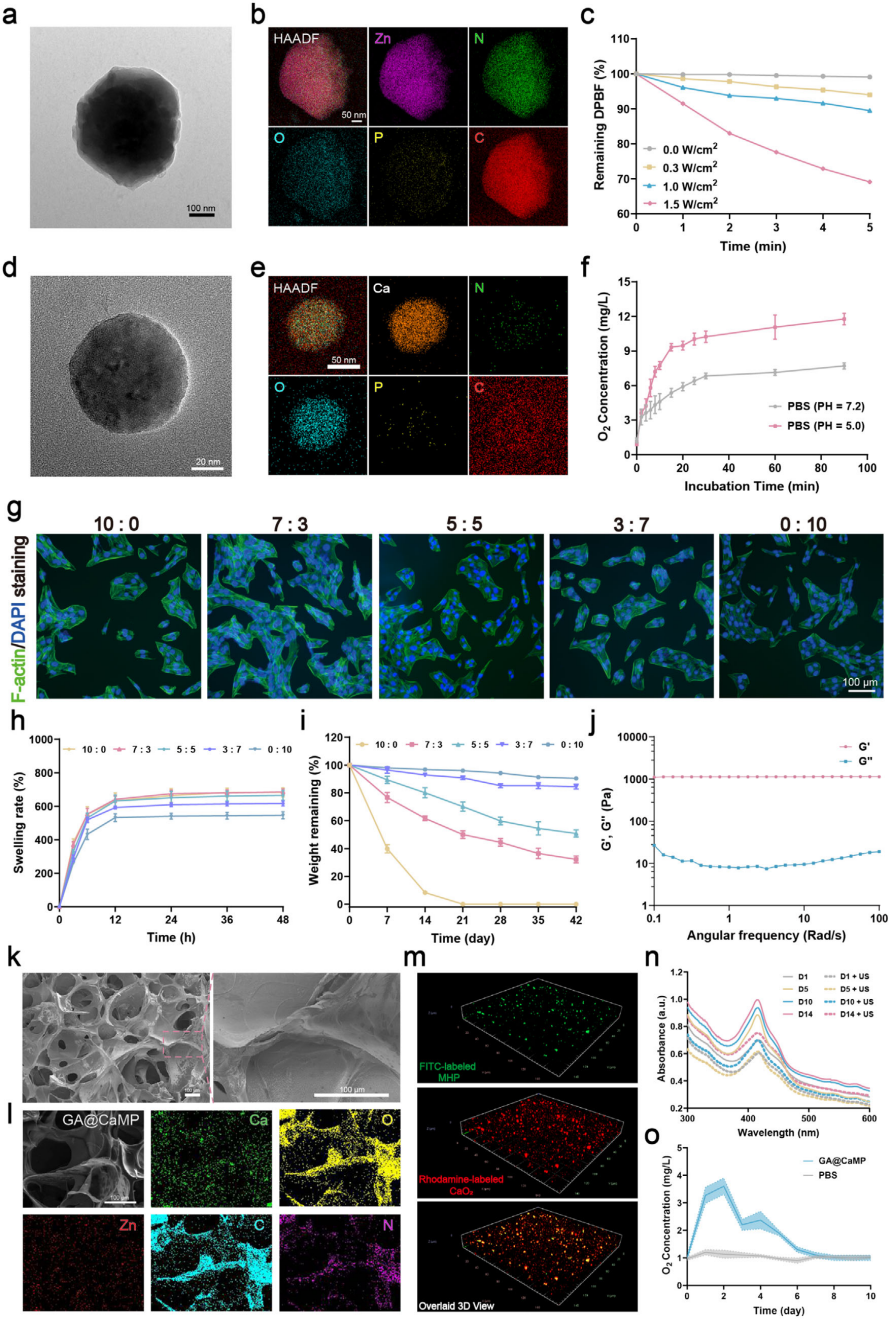
Synthesis and characterization of the GA@CaMP hydrogel system. a) TEM image and b) corresponding elemental mappings of MHP. c) Frequency‐dependent (0, 0.3, 1.0, and 1.5 W cm^−2^) absorbance changes of DPBF co‐incubated with MHP with US. d) TEM image and e) corresponding elemental mappings of CaO_2_ NPs. f) Measurement of O_2_ concentration in PBS (pH 5.0 and 7.2) at a CaO_2_ NPs concentration of 1 mg mL^−1^ (*n* = 3). g) Fluorescence images of the cytoskeletal in BMSCs cultured on GA hydrogels with different compositions (10:0, 7:3, 5:5, 3:7, and 0:10). h) Swelling stability of different GA hydrogels, including 10:0 (yellow), 7:3 (pink), 5:5 (green), 3:7 (purple), and 0:10 (blue), in PBS over 48 h (*n* = 3). i) Degradation curve of different GA hydrogels, including 10:0 (yellow), 7:3 (pink), 5:5 (green), 3:7 (purple), and 0:10 (blue), in PBS containing type II collagenase (2 U mL^−1^) (*n* = 3). j) The elastic (G’) and viscous (G”) moduli of GA hydrogels vary with frequency. k) SEM image and l) element mapping images of GA@CaMP hydrogel. m) Representative CLSM 3D views of dispersibility of nanoparticles in GA@CaMP hydrogel. n) Assessment of ROS generation behavior of the GA@CaMP hydrogel at different US exposure time points (Day 1, 5, 10, and 14). o) Assessment of oxygen release capability of the GA@CaMP system in PBS (pH 5.0) (*n* = 3). Data are presented as Mean ± SD.

The nanomaterial MHP was synthesized via a one‐pot method. In brief, pDNA was first mixed with the Zn^2^⁺ solution, and HMME was added to the 2‐methylimidazole (2‐MIM) solution. Zinc nitrate solution was then added dropwise to the HMME‐doped 2‐MIM solution, resulting in the in‐situ encapsulation of pDNA and HMME in ZIF‐8. This process ensures a more stable encapsulation of the materials, as opposed to relying on the less stable surface adsorption. After centrifugation, a pale pink precipitate, identified as MHP, was obtained. To optimize pDNA loading efficiency and protect it from nuclease degradation, we explored various MHP‐to‐pDNA mass ratios. We performed agarose gel electrophoresis retardation experiments using the material precipitate. The results showed that when the MHP:pDNA mass ratio reached 40:1, pDNA could be effectively complexed (Figure S1, Supporting Information), achieving the highest loading efficiency. We also investigated the optimal loading concentration of HMME. UV–vis absorption spectroscopy revealed that the loading capacity of the nanocarrier approached saturation when 20 µg of HMME was added. Therefore, we selected 20 µg as the loading amount for subsequent experiments to ensure both efficient loading and optimal material utilization (Figure S2a, Supporting Information). At this concentration, the encapsulation efficiency of HMME reached 76.25%, with a loading capacity of 1.39% (Figure S2b, Supporting Information). Transmission electron microscopy (TEM) analysis of ZIF‐8 and MHP revealed that unencapsulated ZIF‐8 exhibited a regular rhombohedral structure with a diameter of approximately 50 nm (Figure S3a, Supporting Information). After encapsulation, the nanoparticles increased in size and exhibited a uniform nanohydrangea structure (**Figures** **2**a and S3b, Supporting Information). Elemental mapping showed a higher phosphorus density in MHP compared to pure ZIF‐8, confirming successful pDNA encapsulation (Figure 2b and Figure S3c, Supporting Information). X‐ray diffraction (XRD) analysis demonstrated that MHP retained the crystalline characteristics of pure ZIF‐8, indicating that pDNA and HMME encapsulation did not significantly affect ZIF‐8 crystallinity (Figure S3d, Supporting Information). Dynamic light scattering (DLS) measurements showed that the average particle size of ZIF‐8 was 53.17 nm, while MHP particles increased to an average of 416.67 nm (Figure S3e, Supporting Information), which was consistent with TEM observations. Zeta potential analysis indicated that the zeta potential of ZIF‐8 decreased from +32.50 to +13.05 mV after encapsulating negatively charged pDNA (Figure S3f, Supporting Information). UV–vis spectroscopy further confirmed HMME encapsulation. Although pure ZIF‐8 displayed a smooth curve, both HMME and MHP exhibited characteristic Q‐band absorption peaks, validating the incorporation of HMME (Figure S3g, Supporting Information). The MHP nanoparticles were incubated in phosphate‐buffered saline (PBS, pH 7.4) for a duration of 2 weeks, during which their hydrodynamic diameter and polydispersity index exhibited no significant variation, thereby confirming their robust colloidal stability under physiological conditions (Figure S3h, Supporting Information).

Subsequently, we evaluated the ROS generation capacity of MHP using 1,3‐diphenylisobenzofuran (DPBF) as an indicator. DPBF can capture singlet oxygen (^1^O_2_), leading to an irreversible decrease in its absorbance at 410 nm. In the presence of prolonged ultrasound (US) irradiation, the DPBF absorbance exhibited a significant decrease (Figure S4a,b, Supporting Information). In the presence of fixed irradiation at 1.0 W cm^−2^, the ROS generation increases roughly proportionally with material concentration (Figure S4c, Supporting Information), and higher US frequency accelerated DPBF degradation (Figure 2c). For instance, at 1.5 W cm^−2^, 30.9% of DPBF was consumed within 5 min, whereas only 8.5% was consumed at 0.3 W cm^−2^ over the same period. We also established a correlation between US frequency and ROS generation, confirming their regulatory relationship (Figures S4d). These results demonstrate that MHP nanoparticles can generate varying amounts of ROS under different US conditions, exhibiting the precise controllability of ROS levels. Given that tumor and bone defect microenvironments are mildly acidic, we selected ZIF‐8 as an acid‐degradable carrier for pDNA. Using a standard curve, we quantified pDNA release from MHP under different pH conditions (Figure S5a,b, Supporting Information). At physiological pH, negligible pDNA release was observed, while at pH 5.0, ZIF‐8 shell degraded gradually, releasing pDNA over time. Notably, significant pDNA release was detected within 30 min, increasing progressively to approximately 50% after 3 h of incubation. These findings confirm the pH‐responsive nature of ZIF‐8 as an effective carrier. To address hypoxia, we synthesized stable calcium peroxide nanoparticles (CaO_2_ NPs) using a previously reported method. CaO_2_ NPs can decompose in acidic environments to release oxygen and calcium ions, making them ideal for osteogenesis. TEM analysis revealed that CaO_2_ NPs were rough‐surfaced spheres with a uniform diameter of approximately 75 nm (Figure 2d and Figure S6a, Supporting Information). DLS measurements showed a hydrated diameter of 98.47 nm, slightly larger than TEM observations (Figure S3e, Supporting Information). Elemental mapping confirmed the spatial distribution of Ca and O within CaO_2_ NPs (Figure 2e). XRD analysis indicated that the crystalline structure of CaO_2_ NPs matched the standard CaO_2_ diffraction pattern (JCPDS no. 003–0865), confirming successful synthesis (Figure S6b, Supporting Information). The oxygen release process of CaO_2_ in aqueous environments proceeds via the following reactions: (1) CaO_2_ + 2H_2_O = Ca(OH)_2_ + H_2_O_2_, (2) 2H_2_O_2_ = 2H_2_O + O_2_↑. When incubated in PBS, CaO_2_ underwent complete degradation within 24 h, resulting in the loss of its initial morphology (Figure S6c, Supporting Information). We further evaluated the oxygen‐releasing capacity of CaO_2_ NPs in PBS at different pH levels. Using a dissolved oxygen microelectrode, we observed that CaO_2_ NPs released oxygen in both conditions, with significantly faster and greater release in mildly acidic environments (Figure 2f). This could be attributed to the fact that in an acidic environment, the high concentration of H⁺ in the solution reacts with Ca(OH)_2_ through a neutralization reaction, continuously driving the reaction forward and significantly increasing the yield of H_2_O_2_. Meanwhile, H⁺ also promotes the decomposition of hydrogen peroxide, further accelerating oxygen release. This strong oxygen‐releasing ability ensures a favorable environment for subsequent bone repair and regeneration.

### 2.2 Preparation and Multifunctional Characterization of GA Hybridized Hydrogel and GA@CaMP Janus Hydrogel Platform

We selected a gelatin methacryloyl/alginate methacrylate (GelMA/AlgMA) composite hydrogel system as the carrier platform. The combination of these two materials leverages the excellent biocompatibility and tunability of GelMA, while taking advantage of AlgMA's ionically crosslinkable properties to enhance the hydrogel's stability and bioactivity.^[^ ^29^, ^30^ ^]^ Both GelMA and AlgMA are functionalized with double bonds, allowing for ultraviolet (UV) light‐induced crosslinking. We first validated the gelation properties of the composite through an inverted bottle experiment. After thoroughly mixing the GelMA and AlgMA solutions, the resulting mixture remained a clear, homogeneous liquid. Upon UV exposure for 5–10 s, the mixture rapidly solidified into a gel (Figure S7a, Supporting Information). Next, we explored the optimal doping ratio of GelMA and AlgMA for bone regeneration scaffolds. Based on previous studies, we controlled the solid content of the hydrogel at 10 wt%.^[^ ^31^, ^32^ ^]^ The detailed mass ratios of GelMA to AlgMA are listed in TableS1 (Supporting Information). We prepared five different hydrogel compositions (GelMA/AlgMA mass ratios of 10:0, 7:3, 5:5, 3:7, and 0:10), along with a hydrogel‐free control group. Bone Marrow Mesenchymal Stem Cells (BMSCs) were seeded on the hydrogels at different ratios, and cell proliferation was observed. Cell Counting Kit‐8 (CCK‐8) assays showed that the cell viability in the hydrogel groups increased with culture time. After 3 days of co‐culture, the 7:3 GA hydrogel group exhibited the highest cell vitality (Figure S7b, Supporting Information). Live/dead staining further confirmed that the cell viability in the hydrogel groups was consistently greater than 90%, indicating excellent cell affinity, with the highest cell density observed on the 7:3 GA hydrogel (Figure S7c, Supporting Information). The 7:3 GA composite hydrogel is likely to optimally simulate the chemical properties and mechanical strength of the extracellular matrix (ECM), providing a favorable microenvironment to support cell growth and tissue repair. To further assess cell adhesion and spreading, we performed cytoskeletal staining. After co‐culturing for 3 days, fluorescence microscopy revealed well‐developed cytoskeletons and cell clusters on the hydrogel surface, with cells displaying good elongation morphology. Among the samples, the 7:3 GA hydrogel facilitated the largest number of cell colonies and the greatest spreading area, promoting superior cell attachment and proliferation (Figure 2g). These findings suggest that the cells maintain a healthy growth state, providing an advantageous environment for subsequent cell proliferation and differentiation.

We further characterized the properties of the hydrogels across different formulations. Initially, the sol–gel transition times were assessed, revealing that all hydrogels exhibited similar gelation profiles, transitioning rapidly to gel within 5–10 s upon UV exposure (Figure S7d, Supporting Information). This characteristic enhances the hydrogel's injectability, making it highly suitable for biomedical applications. We also tested the compressive strength of the hydrogels. At the same solid content, pure AlgMA exhibited the highest compressive strength, while pure GelMA showed the lowest (Figure S7e, Supporting Information). By adjusting the doping ratio, the compressive strength could be tailored to meet the requirements of different environments. The swelling and degradation characteristics of the hydrogels are vital for ensuring the stability of implants and promoting tissue regeneration.^[^ ^33^ ^]^ During the swelling tests, all groups exhibited a rapid increase in swelling during the first 3 h, reaching equilibrium within 48 h. Notably, pure GelMA showed the highest swelling ratio after 48 h, closely followed by the 7:3 GA hydrogel, with a swelling ratio approximately 6.8 times its original dry weight (Figure 2h). As the proportion of AlgMA increased, the swelling ratio gradually decreased, likely due to the increased molecular crosslinking within the hydrogel matrix, which restricts the penetration of solvent molecules into the polymer network. This suggests that a higher swelling ratio correlates with enhanced mass exchange and diffusion capabilities. Finally, we observed the in vitro degradation behavior of the hydrogels. Pure GelMA degraded completely in about 15 days, while the incorporation of AlgMA increased the crosslinking density of the hydrogel matrix, delaying the degradation time of the other groups, showing a trend similar to that of swelling behavior (Figure 2i).

In summary, we selected the 7:3 GA hydrogel as the material carrier. In subsequent experiments, we will refer to it simply as GA. Digital photographs and rheological testing results confirmed the successful formation of the GA hydrogel network structure and its excellent stability (Figure S7f, Supporting Information and Figure 2j). Furthermore, we investigated the effects of applying US at different frequencies on the hydrogel. Scanning electron microscopy (SEM) results demonstrated that US treatment at various frequencies did not disrupt the internal network structure of the hydrogel, ensuring the reliability of the hydrogel for subsequent experiments (Figure S8, Supporting Information).

To construct a sono‐controllable Janus hydrogel platform, MHP and CaO_2_ NPs were incorporated in the GA hydrogels. Based on the results of the CCK‐8 assay, we selected concentrations of 200 µg mL^−1^ for MHP and 600 µg mL^−1^ for CaO_2_ in the hydrogel for subsequent experiments, and designated this system as GA@CaMP (Figure S9a,b, Supporting Information). Rheological tests confirmed the GA@CaMP hydrogel's stability as an elastic solid (Figure S10, Supporting Information). The SEM images of GA@CaMP showed that the encapsulation of the materials did not disrupt the formation of the gel, but the pore size increased, and the pore surface became rougher due to material adhesion (Figure 2k). Elemental mapping images revealed a uniform distribution of C, O, N, Zn, and Ca within GA@CaMP, with Zn originating from MHP nanoparticles, Ca from CaO_2_, and C, O, and N primarily from the hydrogel itself (Figure 2l). Three‐dimensional confocal laser scanning microscopy (CLSM) was employed to further assess the distribution of nanoparticles within the hydrogel. The results revealed that both MHP and CaO_2_ were homogeneously distributed throughout the colloidal network without noticeable aggregation. This homogeneous distribution is critical for ensuring consistent ROS and oxygen levels throughout the hydrogel matrix (Figure 2m). We evaluated the US responsiveness of the hydrogel and found that the GA@CaMP system was able to stably release ROS under US irradiation at different time points (Figure 2n). Subsequently, the oxygen‐releasing capability of the hydrogel was further evaluated. Although CaO_2_ is relatively reactive in aqueous solutions, its encapsulation within the GA hydrogel enabled sustained oxygen release for about one week (Figure 2o). In addition, intracellular oxygen levels were monitored by measuring the fluorescence intensity of Ru(dpp)_2_Cl_2_. Notably, treatment with the CaO_2_‐loaded hydrogel resulted in a significant decrease in fluorescence intensity in hypoxia‐treated cells, indicating effective in vitro oxygenation (Figure S11, Supporting Information).

### 2.3 In Vitro Cytotoxicity, Internalization, and Antitumor Efficacy of MHP in 4T1 Breast Cancer Cells

**Figure 3:**
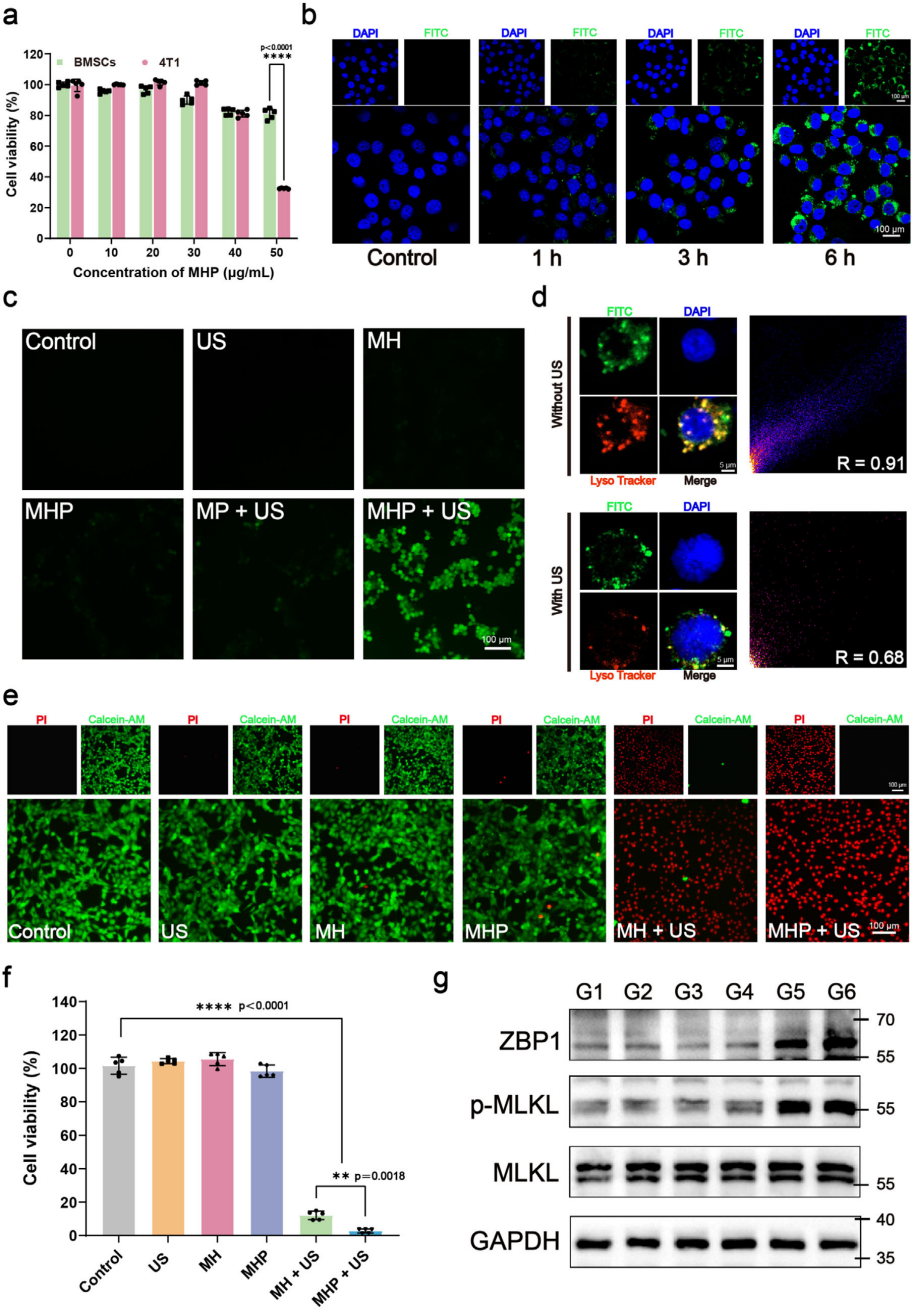
Assessment of MHP's anticancer impact on 4T1 cells in vitro. a) The relative viability of 4T1 cells and BMSCs co‐cultured with varying MHP concentrations (0, 10, 20, 30, 40, and 50 µg mL^−1^) for 24 h (*n* = 5). b) CLSM images of FITC‐labeled MHP uptake by 4T1 cells at different time points (0, 1, 3, and 6 h). c) Fluorescence images showing ROS levels in 4T1 cells. d) Representative CLSM images and corresponding confocal imaging study of 4T1 cells co‐stained with LysoTracker Red and FITC‐labeled MHP, with or without US. *R* represents the Pearson correlation coefficient. e) Fluorescence images of live/dead staining and **f)** relative cell viabilities of 4T1 cells after treatments, including Control, US only, MH, MHP, MH + US, and MHP + US (*n* = 5). g) Western blotting analysis of ZBP1, MLKL, and p‐MLKL protein expression levels in 4T1 cells after different treatments, including Control, US only, MH, MHP, MH + US, and MHP + US. Data are presented as Mean ± SD. Significance between multiple groups was calculated using one‐way ANOVA and Tukey–Kramer multiple comparisons test. ****P < 0.0001, ***P < 0.001, **P < 0.01, *P < 0.05, ns: no significance.

Prior to evaluating the antitumor efficacy, we assessed the cellular safety of MHP nanoparticles using the CCK‐8 assay. After a 24 h incubation, significant growth inhibition was observed in 4T1 cells at an MHP concentration of 50 µg mL^−1^, while BMSCs maintained good cellular viability (**Figure** **3**a). Next, we used CLSM to observe the internalization of MHP nanoparticles at different time points. The observation showed that after 1 h of co‐incubation with 4T1 cells, a small amount of MHP was internalized. Over time, the engulfment of MHP significantly increased (Figure 3b). A similar increase in engulfment over time was observed in BMSCs (Figure S12, Supporting Information). Efficient lysosomal/endosome escape is crucial for gene delivery. On one hand, the proton sponge effect mediated by the imidazole ring ligands of MHP helps reduce degradation within lysosomes.^[^ ^27^ ^]^ On the other hand, the ROS generated under US exposure can disrupt endosomes/lysosomes, thereby enhancing gene delivery efficiency.^[^ ^34^ ^]^ Before investigating the lysosomal escape ability of the materials, we first assessed the ROS generation capability of these materials under US and non‐US conditions. Fluorescence microscopy images showed negligible ROS generation without US exposure. However, only under US exposure did MHP generate a large amount of ROS, as indicated by bright green fluorescence, which originates from the encapsulated HMME component (Figure 3c). It has been reported that ZIF‐8, as a “protective shell,” enhances the stability of HMME and improves the generation efficiency of ^1^O_2_.^[^ ^27^ ^]^ Subsequently, we used CLSM to observe the localization of MHP nanoparticles within the organelles. In both 4T1 and BMSC cells, the addition of US promoted the separation of the green fluorescence (from FITC‐labeled MHP) and the red fluorescence (from lysosomes). Pearson correlation analysis further confirmed the effective lysosomal/endosomal escape of MHP (Figure 3d and Figure S13, Supporting Information). The efficient uptake and delivery of MHP nanoparticles is a key prerequisite for successful plasmid expression. Next, we explored the in vitro antitumor effects and mechanism of the MHP nanocarrier system on 4T1 cells. We evaluated cellular activity at different US frequencies, with the most potent tumor‐killing effect observed at a US frequency of 1.5 W cm^−2^ (Figure S14, Supporting Information). Calcein‐AM/PI staining revealed that the MH + US and MHP + US groups showed significantly higher numbers of propidium iodide (PI)‐positive dead cells, with much stronger red fluorescence than in the other groups (Figure 3e and Figure S15, Supporting Information). Consistent results were observed in the CCK‐8 assay, where the average cell death rate in the MHP + US group reached 97.2% for 4T1 tumor cells (Figure 3f). This indicates that the MHP nanocarrier system, assisted by US, exhibits strong antitumor efficacy. Notably, cell viability in the MHP + US group was lower than that in the MH + US group. This may be due to the transfection of the ZBP1 plasmid, which triggers the activation of the necroptosis pathway in 4T1 cells.^[^ ^35^ ^]^ ZBP1, as a nucleic acid sensor (primarily Z‐RNA), can recognize nucleic acids with abnormal conformations, thereby triggering necroptosis in cells.^[^ ^25^, ^35^ ^]^ Western blotting (WB) analysis was used to investigate the ZBP1 transfection and necroptosis induction in 4T1 cells treated with MHP + US. Phosphorylation of MLKL is a key step in the execution of necroptosis. WB analysis revealed that, compared to the other groups, the MHP + US‐treated 4T1 cells showed a significant upregulation of both ZBP1 and p‐MLKL, indicating that the successful transfection of ZBP1 effectively activated the necroptosis process in tumor cells, leading to efficient tumor cell killing (Figure 3g and Figure S16a, Supporting Information). Meanwhile, we examined the activation status of the Wnt/β‐catenin pathway and found that the expression levels of β‐catenin protein showed no significant changes among the groups (Figure S16b, Supporting Information). This suggests that ZBP1‐induced necroptosis does not markedly suppress the Wnt/β‐catenin pathway to inhibit cell proliferation, but rather may directly terminate cellular activity by disrupting the cell membrane structure and inducing the release of intracellular contents. We also compared the activation of the necroptotic pathway in 4T1 cells when applying US at different intensities (0.3 and 1.5 W cm^−2^). The results demonstrated that irradiation with low‐intensity US (0.3 W cm^−2^) failed to activate the necroptotic pathway, whereas high‐intensity US (1.5 W cm^−2^) significantly triggered this pathway (Figure S16c, Supporting Information). Existing studies have shown that oxidative stress can trigger endogenous Z‐form nucleic acids, thereby inducing ZBP1 activation in a clear dose‐ and time‐dependent manner.^[^ ^36^ ^]^ Consistently, when 4T1 cells were exposed to US at 1.5 W cm^−2^, the substantial ROS generation effectively activated ZBP1‐mediated necroptosis. In contrast, the small amount of ROS produced under low‐intensity US (0.3 W cm^−2^) was insufficient to reach the threshold required to initiate necroptosis.

### 2.4 In Vivo Evaluation of GA@CaMP Hydrogel for Antitumor Effects and Immune Activation

Before assessing the therapeutic effects of GA@CaMP in vivo, we first verified the safety of the sono‐controllable Janus hydrogel platform. Compared to the PBS group, there were no significant changes in hematological, biochemical, and hepatorenal function indicators in the GA@CaMP and GA@CaMP + US groups. Additionally, there were no notable differences in the body weight of the mice across the three groups (Figure S17a–c, Supporting Information). The major organs of mice in all groups showed no apparent signs of inflammation or damage (Figure S17d, Supporting Information). We then used small animal in vivo imaging systems (IVIS) to monitor the in vivo release profile of GA@CaMP in mice. On day 0, the entire gel exhibited strong fluorescence. Over time, MHP was gradually released, and the fluorescence intensity around the gel diminished, with the fluorescent signal shrinking in range. However, on day 20, a faint MHP accumulation signal was still detectable within the gel, indicating the platform's sustained release capacity and significantly enhanced drug infiltration at the tumor site (Figure S18, Supporting Information).

**Figure 4:**
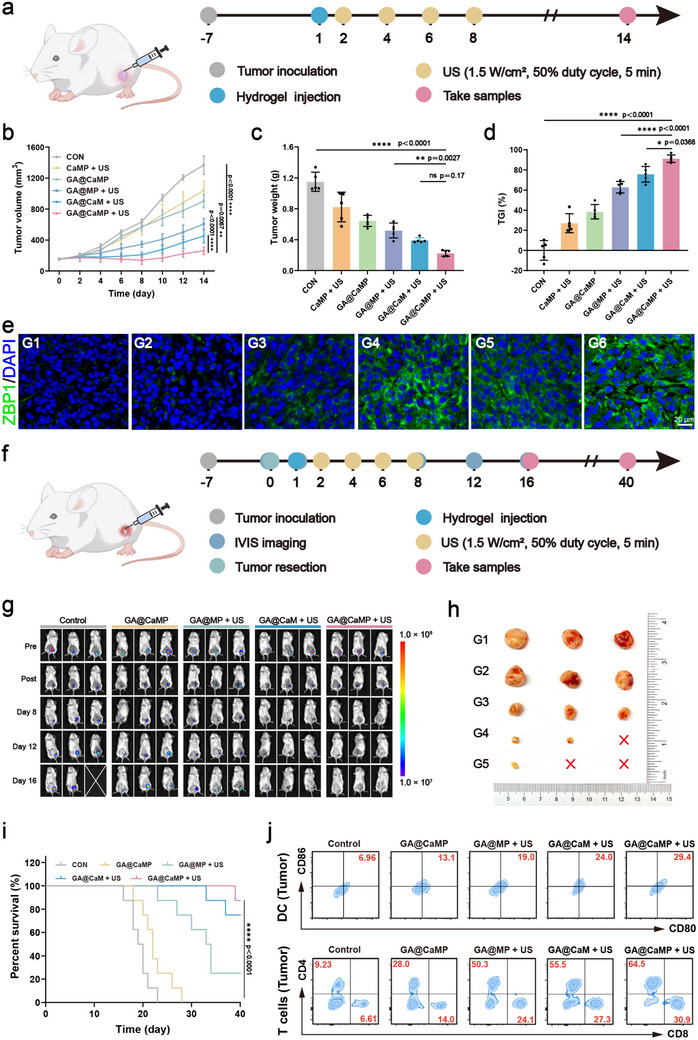
The effectiveness of antitumor performance of GA@CaMP in vivo. a) Schematic illustration of GA@CaMP hydrogels for inhibiting tumor growth in the in situ 4T1 tumor model (Control, CaMP + US, GA@CaMP only, GA@MP + US, GA@CaM + US, GA@CaMP + US). b) Average tumor growth curves (*n* = 5). c) Average weight of excised tumors and d) tumor‐inhibition ratios in the 4T1 tumor model after treatments (*n* = 5). e) Immunofluorescent staining of ZBP1 in tumor tissues after various treatments (G1: CON, G2: CaMP + US, G3: GA@CaMP only, G4: GA@MP + US, G5: GA@CaM + US, G6: GA@CaMP + US). f) Schematic illustration of GA@CaMP hydrogel for inhibiting 4T1 tumor recurrence in an incomplete tumor resection model (Control, GA@CaMP only, GA@MP + US, GA@CaM + US, GA@CaMP + US). g) Bioluminescence images of tumor‐bearing BALB/c mice at different times (Pre, Post, Day 8, Day 12, and Day 16) after various treatments, including Control, GA@CaMP only, GA@MP + US, GA@CaM + US, GA@CaMP + US (*n* = 3). h) Photographs of the tumors collected from postoperative recurrent tumor models after 16 days of different treatments (G1: Control, G2: GA@CaMP only, G3: GA@MP + US, G4: GA@CaM + US, G5: GA@CaMP + US) (*n* = 3). i) Survival curves of 4T1 tumor‐bearing mice with different treatments, including Control, GA@CaMP only, GA@MP + US, GA@CaM + US, GA@CaMP + US (*n* = 8). j) Typical flow cytometry of mature DCs, CD4^+^ and CD8^+^ T cells in the recurrent tumors after different treatments, including Control, GA@CaMP only, GA@MP + US, GA@CaM + US, GA@CaMP + US. Data are presented as Mean ± SD. Significance between multiple groups was calculated using one‐way ANOVA and Tukey–Kramer multiple comparisons test. Kaplan–Meier survival analysis was performed to assess animal survival status, and the log‐rank test was used to determine the significance of differences in survival between groups. ****p < 0.0001, ***p < 0.001, **p < 0.01, *p < 0.05, ns: no significance.

Next, we evaluated the in vivo antitumor effect of GA@CaMP. The treatment protocol is shown in **Figure** **4**a. During the entire treatment period, the body weight of mice across all groups fluctuated minimally with no significant differences (Figure S19a, Supporting Information). Compared to the CON group, the GA@CaMP + US group showed a marked inhibition of tumor growth (Figure 4b and Figure S19b, Supporting Information). Digital photos of the resected tumors showed that the GA@CaMP + US group had the smallest tumor volume and weight compared to the other groups (Figure S19c and Figure 4c), with a tumor growth inhibition (TGI) rate of 91.2 ± 3.7% (Figure 4d). Without the hydrogel, a significant tumor‐killing effect was observed immediately after the first US irradiation; however, the inhibitory effect was no longer significant in subsequent treatments. The presence of the hydrogel significantly prolongs the retention time of the material within the tumor, effectively achieving a controlled‐release effect. Additionally, we observed that both the tumor size and TGI (62.8 ± 5.8%) in the GA@MP + US group, which lacked CaO_2_, were significantly lower than those in the GA@CaMP + US group (Figure 4b–d). Immunohistochemical staining also revealed that the expression of HIF‐1α in tumor tissues was significantly reduced to varying degrees in the treatment groups containing CaO_2_ (Figure S19d, Supporting Information). This suggests that alleviating the hypoxic environment can effectively enhance the efficiency of cell killing. Immunofluorescence staining of ZBP1 expression showed that in the CON group, the expression of ZBP1 was very low. After plasmid supplementation, the expression level increased, with the GA@CaMP + US group showing the most prominent green fluorescence, indicating successful transfection and expression of the ZBP1 plasmid in tumor cells (Figure 4e). Furthermore, histological analysis with hematoxylin and eosin (H&E) staining and Ki67 staining demonstrated that the GA@CaMP + US group exhibited the strongest antitumor effect and effectively suppressed tumor cell proliferation (Figure S19e,f, Supporting Information). To elucidate the therapeutic mechanism of GA@CaMP, we evaluated the intracellular ROS generation. The fluorescence intensity of ROS in the GA@CaMP + US treatment group was significantly higher than that in the other groups (Figure S20, Supporting Information). This further confirms that the supplementation of oxygen, in synergy with ZBP1, disrupts the redox homeostasis and effectively promotes ROS generation, thereby enhancing the anti‐cancer efficacy. After treatment, major organs from the euthanized mice were collected and subjected to H&E staining, with no significant tissue inflammation or apoptosis observed (Figure S21, Supporting Information), further confirming the safety of the treatment approach.

In addition to systemic treatments, surgery is a mainstay treatment for breast cancer with bone metastasis. However, due to the infiltrative nature and location of the lesions, there is often a risk of incomplete excision and subsequent recurrence. We established a partial tumor resection model to further explore the inhibitory effect of GA@CaMP sono‐controllable Janus hydrogel platform on local recurrence of residual tumor cells. The treatment protocol is shown in Figure 4f. IVIS detection revealed that in the GA@CaMP + US group, the tumor signal intensity dropped to undetectable levels after the US treatment, and no significant recurrence signal was detected in the following 8 days. In contrast, significant recurrence signals were observed in the other four groups within the observation period (Figure 4g and Figure S22a, Supporting Information). On day 16, resected tumors from recurrent sites showed that the GA@CaMP + US group had the fewest recurrent tumors, the smallest volume, and significantly reduced weight compared to the CON group (Figure 4h and Figure S22b, Supporting Information). Thanks to its excellent effect in inhibiting recurrence, the GA@CaMP + US group showed significantly extended survival, with a survival rate of 87.5% over 40 days (Figure 4i). In the GA@MP + US group lacking CaO_2_, the mouse survival rate was only 25%. The enhanced oxygen supply augmented the sonodynamic effect, inhibited the progression of recurrent tumors at an early stage, and significantly prolonged the survival time of mice.

We sought to determine whether the remarkable antitumor effects of the GA@CaMP hydrogel platform under US irradiation were linked to immune system activation. After 14 days of treatment, tumors from the different treatment groups were harvested, and immune cell responses and the expression of major immune factors within the tumors were assessed. Flow cytometry (Figure S23, Supporting Information, shows the gating strategy) and quantitative results showed that the proportion of mature DCs in the GA@CaMP + US group was significantly higher than in the other groups, averaging 31.3 ± 1.7% (Figure 4j and Figure S24a, Supporting Information). Additionally, the infiltration of cytotoxic T cells (CD8^+^ cells) in the tumor was significantly enhanced after GA@CaMP + US treatment, being 5.2 times greater than in the CON group (Figure 4j and Figure S24b, Supporting Information). A notable increase in helper T cells (CD4^+^ cells) was also observed, with their numbers increasing 4.9‐fold compared to the CON group (Figure S24c, Supporting Information). Immunofluorescence staining of tumor sections confirmed the significantly increased infiltration of CD4^+^ cells and CD8^+^ cells, consistent with the flow cytometry results (Figure S24d, Supporting Information). Interestingly, in the GA@CaM + US group without the ZBP1 plasmid, although the infiltration of mature DC and CD8^+^ cells was significantly increased, it was still lower than that observed in the GA@CaMP + US group, indicating that ZBP1 contributes to immune activation. This may be due to ZBP1 triggering MLKL‐dependent necroptosis, which releases inflammatory signaling molecules and intracellular tumor antigens, thereby inducing a potent immune response.^[^ ^24^, ^25^ ^]^ Finally, we evaluated tumor immune markers and found that in the GA@CaMP + US group, typical pro‐inflammatory cytokines, including interleukin‐6 (IL‐6), interleukin‐12p70 (IL‐12p70), tumor necrosis factor‐alpha (TNF‐α), and interferon‐gamma (IFN‐γ), were significantly elevated within the tumor (Figure S24e, Supporting Information). Overall, these results confirm that a sono‐controllable Janus hydrogel platform therapeutic strategy can reshape the immunosuppressive microenvironment, effectively activate immune cell infiltration and the expression of specific immune factors, thereby inhibiting tumor growth and recurrence.

### 2.5 Evaluation of GA@CaMP Hydrogel in Inhibiting Breast Cancer Bone Metastasis and Exploring the Underlying Biological Mechanisms

**Figure 5:**
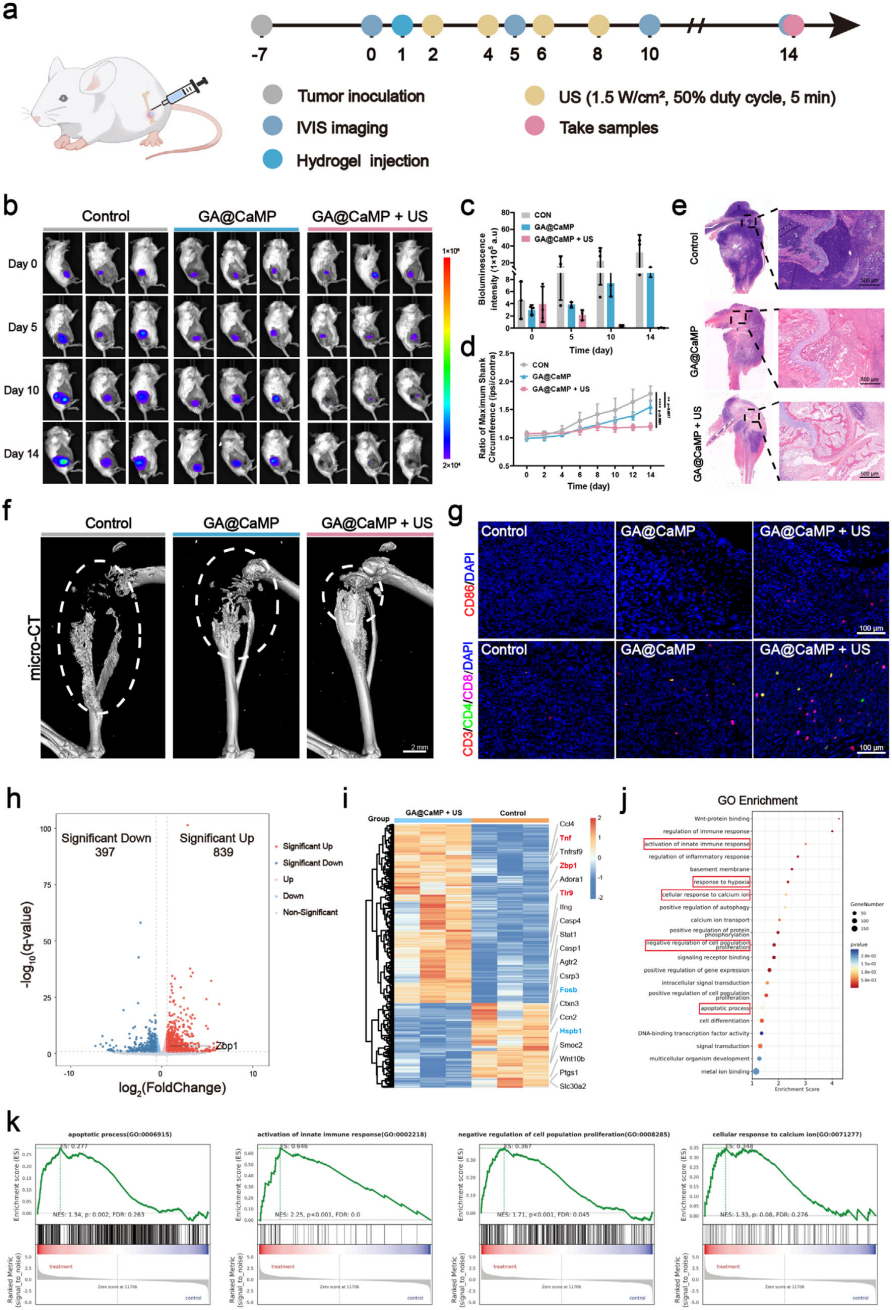
Analysis of the antitumor effect and potential mechanism of GA@CaMP + US in model mice with bone metastases (Control, GA@CaMP, GA@CaMP + US). a) Schematic illustration of GA@CaMP hydrogels for inhibiting tumor growth in a bone metastases model. b) Bioluminescence images and c) corresponding fluorescence intensities of BALB/c mice with bone metastases at different time points (Day 0, Day 5, Day 10, and Day 14) after various treatments (Control, GA@CaMP, GA@CaMP + US) (*n* = 3). d) Ratio of maximum shank circumference (ipsilateral/contralateral) (*n* = 3). e) H&E staining images and f) micro‐CT reconstruction images of the metastatic tumor‐bearing tibiae. g) Immunofluorescence images of proliferated mature DCs, CD8^+^ T cells, and CD4^+^ T cells in bone metastases tumor tissue slices (Control, GA@CaMP, and GA@CaMP + US). h) Volcano plots of gene alteration in the GA@CaMP + US group compared to the Control group (*n* = 3). i) Cluster diagram of differentially expressed genes (DEGs) between the GA@CaMP + US group and the Control group (*n* = 3). j) Results of gene ontology (GO) enrichment analysis of potential enrichment pathways after GA@CaMP + US treatments. k) Gene set enrichment analysis (GSEA) of tumors isolated from Control and GA@CaMP + US‐treated mice. Data are presented as Mean ± SD. Significance between multiple groups was calculated using one‐way ANOVA and Tukey–Kramer multiple comparisons test. ****p < 0.0001, ***p < 0.001, **p < 0.01, *p < 0.05, ns: no significance.

Finally, we used luciferase‐labeled 4T1 cells to inoculate the right tibial plateau of mice, simulating a breast cancer bone metastasis model, to further evaluate the anti‐metastatic tumor activity of the GA@CaMP sono‐controllable Janus hydrogel platform and verify its reliability. The treatment process is illustrated in **Figure** **5**a. Unsurprisingly, the mice in the GA@CaMP + US group exhibited a significant reduction in the fluorescence intensity of tumor cells starting on day 5, indicating a significant inhibition of tumor growth. By day 14, some mice showed undetectable tumor signals (Figure 5b,c). At the end of the treatment period, the GA@CaMP + US group showed a marked reduction in metastatic tumor volume and weight compared to the control group and the GA@CaMP group (Figure 5d and Figure S25a,b, Supporting Information). Notably, the GA@CaMP group alone also exhibited a certain degree of tumor‐inhibitory effect. After harvesting the tibiae of the mice, H&E staining was performed. The tumors in the Control group were active and highly invasive, almost completely eroding the bone tissue. In contrast, bone resorption in the GA@CaMP + US group was significantly suppressed, and normal structural units of bone tissue remained intact (Figure 5e). Micro‐CT analysis of the reconstructed images of the tibiae from the breast cancer bone metastasis mice revealed that the Control group exhibited significant expansile bone destruction, cortical continuity disruption, partial bone dissolution, and bone spurs at the edges. By comparison, the extent of bone damage was markedly alleviated in the other treatment groups, especially in the GA@CaMP + US group. The GA@CaMP + US group significantly reduced bone resorption in the tumor‐bearing tibiae, with the bone tissue remaining intact and the bone cortex relatively smooth, showing only minimal bone destruction (Figure 5f). These results demonstrate that the GA@CaMP gel under US treatment can significantly inhibit the spread and invasion of metastatic tumors while protecting the structural integrity of bone tissue. We further investigated immune cell responses within metastatic bone tumors under different treatments. The results showed that, compared to the Control group, the GA@CaMP group alone activated a subset of immune cells. Notably, the GA@CaMP + US group exhibited a significant increase in the infiltration of mature DCs, CD4^+^ cells, and CD8^+^ cells within the tumor microenvironment (Figure 5g). These findings further corroborate the synergistic advantage of this comprehensive therapeutic strategy, which incorporates immunotherapy. Given that bone metastases facilitate secondary tumor spread to the lungs, accelerating cancer progression, we examined lung tissues at the end of treatment to assess distant metastasis. The number of metastatic nodules in the lungs of mice treated with GA@CaMP + US was significantly lower than that of the Control group (Figure S26, Supporting Information). These results indicate that, without timely intervention, bone metastatic tumors can rapidly grow and invade the lungs via the circulatory system, significantly compromising patient quality of life and survival. Here, we propose a promising therapeutic approach that not only effectively inhibits metastatic tumor growth and preserves bone tissue integrity but also suppresses the development of secondary metastasis, offering hope for patients with breast cancer bone metastasis.

To explore the underlying biological mechanisms by which GA@CaMP + US inhibits bone metastatic tumor development, we randomly selected 3 mice from the control group and 3 from the treatment group (GA@CaMP + US), extracted RNA from their tumor tissues, and performed transcriptome sequencing to investigate gene alterations post‐treatment. The sequencing results revealed a total of 1236 differentially regulated genes. Compared to the Control group, 839 genes (67.88%) were upregulated, and 397 genes (32.12%) were downregulated in the treatment group (*P* < 0.05, |log_2_FoldChange | > 0.58) (Figure 5h and Figure S27a, Supporting Information). Among these, ZBP1 was upregulated (FoldChange = 1.96, *p* < 0.00001), further confirming the successful delivery of the ZBP1 plasmid to tumor cells. The heatmap revealed that aside from the upregulated ZBP1, most of the highly upregulated differentially expressed genes are associated with inflammation and immune responses, including the pro‐inflammatory cytokine TNF‐α and the innate immune response receptor TLR9, which activates the immune system. Correspondingly, we also noted that several genes associated with tumor cell proliferation and invasion were significantly downregulated, such as Fosb and Hspb1 (Figure 5i). ZBP1‐mediated necroptosis represents a highly inflammatory form of cell death, characterized by cell swelling and subsequent onclysis. The disintegrated cells then release a variety of signaling molecules, collectively referred to as damage‐associated molecular patterns (DAMPs), which trigger a strong immune response. Compared with apoptosis, which is generally non‐inflammatory and immunologically silent, necroptosis is much more potent in triggering inflammatory and adaptive immune responses.^[^ ^37^ ^]^ The results of the heatmap also confirm that our treatment effectively activates immunity within metastatic tumors and inhibits the proliferation and invasion of tumor cells. Gene Ontology (GO) analysis indicated that these differential genes were closely associated with multiple signaling pathways, including tumor immune activation, responses to calcium ions and hypoxic microenvironments, cell proliferation inhibition, and apoptosis (Figure 5j). Notably, the GO‐derived chord diagram shows that ZBP1 participated in two biological pathways, innate immune activation and apoptosis, in the treatment group, consistent with previous reports (Figure S27b, Supporting Information).^[^ ^23^, ^24^, ^25^ ^]^ cGAS is an intracellular DNA sensor that recognizes cytosolic DNA and activates the STING signaling pathway, initiating innate immune responses and tumor suppression. ZBP1, an important downstream effector molecule in the cGAS‐STING pathway, promotes tumor resistance to DNA damage and strengthens immune suppression when expressed at low levels, especially in triple‐negative breast cancer. In addition, unlike classical apoptosis, ZBP1 induces programmed necrosis, dependent on signaling proteins such as RIPK3 and MLKL, which play a significant role in inhibiting tumor cell proliferation. Subsequently, we performed Gene Set Enrichment Analysis (GSEA) to generate scores for immune and apoptosis‐related pathways. Poptosis and innate immune response activation pathways were significantly upregulated in the tumors of the treatment group, while pathways related to cellular response to calcium ions and negative regulation of cell proliferation were also widely enriched in the post‐treatment tumors (Figure 5k). Similarly, a KEGG analysis provided comparable results, revealing that the differential genes were enriched in multiple signaling pathways, mainly including antigen processing and presentation, Toll‐like receptor (TLR), and Nod‐like receptor (NLR) signaling, further confirming the close correlation between GA@CaMP + US treatment and activation of the innate immune system.^[^ ^38^ ^]^ In addition, consistent with the GO analysis, the calcium ion signaling pathway was also significantly upregulated (Figure S27c,d, Supporting Information). Calcium ion plays a multifaceted role in cancer therapy. Under high ROS levels, Calcium ions can synergistically enhance antitumor efficacy. Additionally, it modulates the tumor microenvironment and strengthens antitumor immune responses.^[^ ^39^ ^]^ Beyond its role in cancer treatment, calcium is an essential element for bone regeneration, as it promotes osteoblast proliferation and differentiation, accelerates mineralization, and regulates inflammation.^[^ ^21^ ^]^ Our Janus hydrogel platform incorporates CaO_2_ as a calcium reservoir, facilitating these therapeutic effects.

### 2.6 In Vitro Validation of GA@CaMP Hydrogel for Osteogenic Differentiation of BMSCs

**Figure 6:**
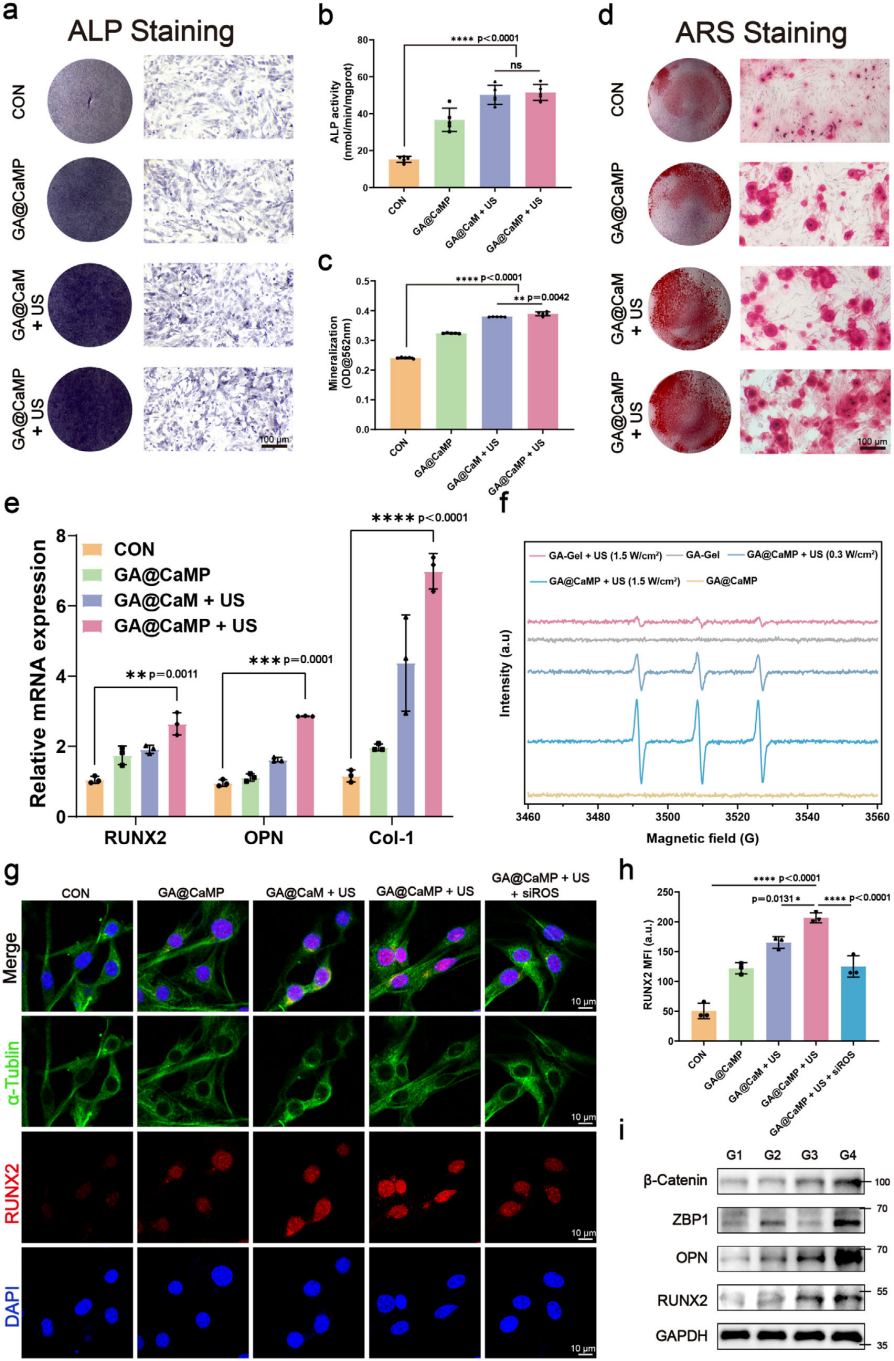
The effectiveness of osteogenic differentiation of BMSCs cultured with GA@CaMP. a) Macroscopic images of ALP staining of BMSCs. b) ALP activity of BMSCs cultured for 7 days (*n* = 5). c) Quantitative analysis of cell mineralization after 14 days of culture (*n* = 5). d) Macroscopic images of ARS staining of BMSCs. e) Relative mRNA expression of osteogenesis‐related genes in BMSCs, including RUNX2, OPN, and Col‐1 (*n* = 3). f) ESR spectra of ^1^O_2_ trapped by TEMP after treatments. g) Immunofluorescence staining and h) quantitative analysis of RUNX2 in BMSCs after various treatments, including Control, GA@CaMP only, GA@CaM + US, GA@CaMP + US, and GA@CaMP + US + siROS (*n* = 3). i) Western blotting analysis of osteogenesis‐related proteins and ZBP1 protein expression in BMSCs after various treatments, including Control, GA@CaMP only, GA@CaM + US, and GA@CaMP + US. Data are presented as Mean ± SD. Significance between multiple groups was calculated using one‐way ANOVA and Tukey–Kramer multiple comparisons test. ****p < 0.0001, ***p < 0.001, **p < 0.01, *p < 0.05, ns: no significance.

Osteogenic differentiation of BMSCs is a prerequisite for bone repair. The effect of the gel platform on osteogenic differentiation of BMSCs was evaluated using the Transwell system. Initially, the safety of BMSCs under varying US exposure times was assessed. The CCK‐8 assay demonstrated that US irradiation within 15 min had no adverse effect on BMSCs viability (Figure S28, Supporting Information). Alkaline phosphatase (ALP) staining and quantitative assays were performed to assess early osteogenic activity and functional status of BMSCs. After 7 days of co‐culture, the GA@CaMP + US group exhibited the darkest ALP staining, followed by the GA@CaM + US and GA@CaMP groups (**Figure** **6a**). Quantitative analysis of ALP activity showed that the ALP activity in the GA@CaM + US, GA@CaMP, and GA@CaMP + US groups was significantly higher than that in the control group (Figure 6b). Alizarin Red S (ARS) staining was used as an important indicator of late‐stage osteogenic differentiation to assess the ability of cells to form mineralized matrices by calcium mineral deposition. After 14 days of co‐culture, groups subjected to US treatment exhibited significantly more red calcium nodules, with quantitative results showing enhanced positive staining, indicating an increase in mineral deposition during bone induction (Figure 6c,d). Even the pure hydrogel group exhibited higher mineralized nodule staining compared to the control group. Notably, the ARS staining in the GA@CaMP + US group was slightly higher than that in the GA@CaM + US group. Studies have shown that ZBP1 plays a role in bone metabolism and can regulate the Wnt/β‐catenin signaling pathway to promote osteogenic differentiation of mesenchymal stem cells, making it an important factor in regulating osteogenesis.^[^ ^26^ ^]^ Next, we aimed to further investigate the mechanism by which GA@CaMP promotes osteogenic differentiation under low‐frequency US. Real‐time quantitative polymerase chain reaction (RT‐qPCR) results revealed that the GA@CaMP + US group exhibited significantly higher mRNA expression levels of RUNX2, OPN, and Col‐1 than the CON group, demonstrating the strongest upregulation of osteogenic markers (Figure 6e). To demonstrate whether the minimal amounts of ROS induced by low‐frequency US play a role in osteogenic differentiation, we assessed the ROS production in BMSCs. However, due to the minimal ROS generation, no ROS signal was detected via immunofluorescence (Figure S29, Supporting Information). More sensitive electron spin resonance (ESR) spectroscopy confirmed the generation of ^1^O_2_ upon GA@CaMP under low‐frequency US (Figure 6f). Additionally, varying US frequencies resulted in different amounts of ^1^O_2_ production, further verifying the reliable sonodynamic properties of the GA@CaMP gel platform and its excellent tunability. Subsequently, we neutralized ROS. Immunofluorescence results showed a significant reduction in osteogenic‐related protein expression in BMSCs after ROS neutralization (Figure 6g,h, and Figure S30, Supporting Information). Numerous studies have shown that low concentrations of ROS, acting as molecular signals, activate an oxidoreductive microenvironment that induces defense signaling, thereby regulating and influencing various cellular processes.^[^ ^12^, ^40^ ^]^ Our study further supports the notion that low‐concentration ROS‐mediated oxidoreductive microenvironments promote bone repair and regulation.^[^ ^12^ ^]^ WB analysis was performed to evaluate the expression levels of ZBP1 and osteogenesis‐related proteins. In the GA@CaMP + US group, the expression levels of ZBP1, RUNX2, and OPN proteins were significantly elevated, while β‐catenin expression also increased accordingly (Figure 6i and Figure S31a, Supporting Information). Studies have shown that either low concentrations of ROS or upregulation of ZBP1 can activate the Wnt/β‐catenin signaling pathway, promoting osteogenesis. Here, we successfully employed the sono‐controllable Janus hydrogel platform to synergistically enhance both factors, thereby accelerating bone repair and regeneration. Meanwhile, we examined the status of the necroptosis pathway in each group and found that in the GA@CaMP + US group, where the Wnt/β‐catenin signaling pathway was significantly activated, the expression level of p‐MLKL was markedly reduced (Figure S31b, Supporting Information). This result suggests that in BMSCs, an active Wnt pathway may exert an inhibitory effect on MLKL phosphorylation, thereby decreasing the cells’ susceptibility to necroptosis.

### 2.7 Evaluation of GA@CaMP Hydrogel for Bone Regeneration in a Critical‐Size Cranial Defect Model

**Figure 7:**
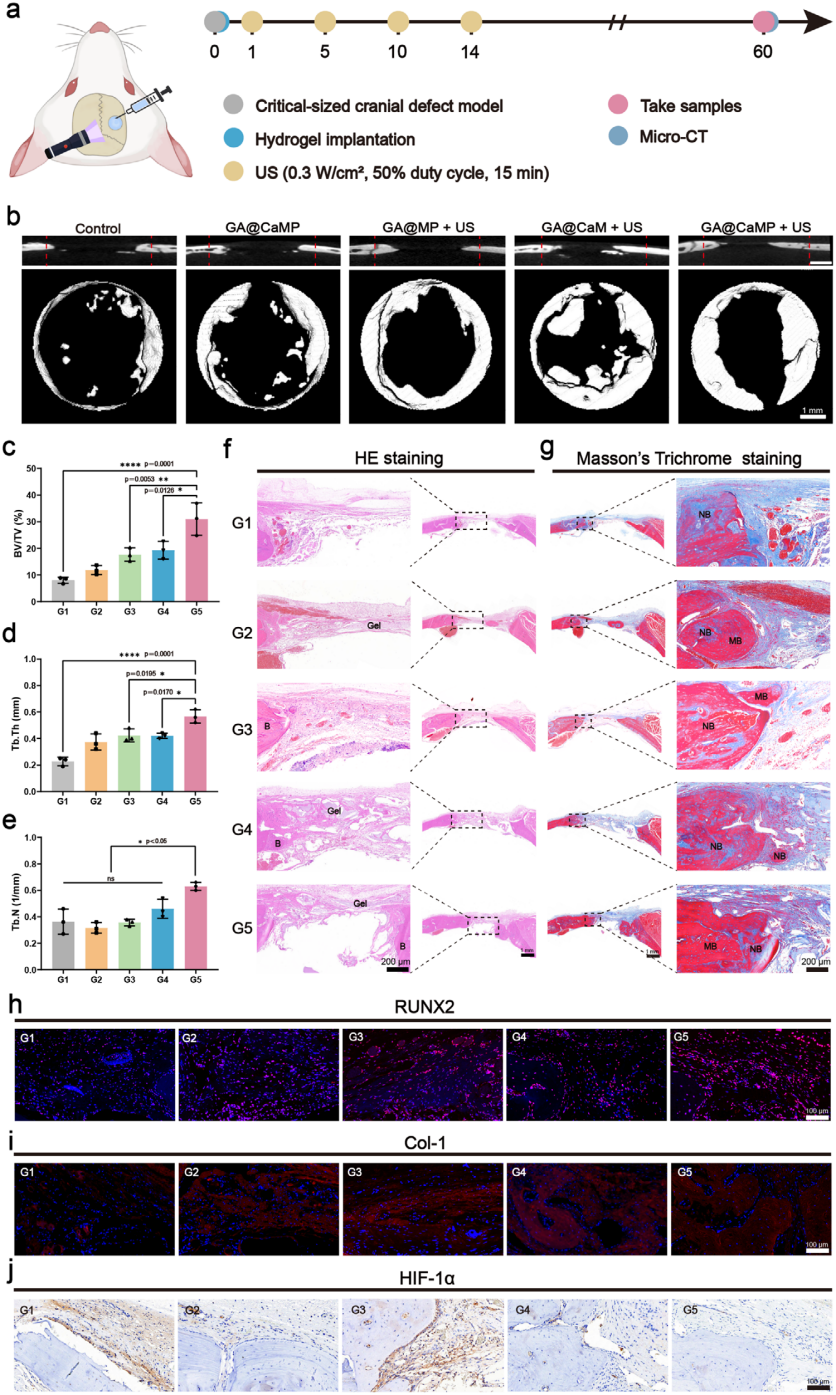
Assessment of the bone repair efficacy of GA@CaMP in rats with skull defect models (Control, GA@CaMP only, GA@MP + US, GA@CaM + US, GA@CaMP + US). a) Schematic illustration of GA@CaMP hydrogels for bone repair in a cranial defect mode (Control, GA@CaMP only, GA@MP + US, GA@CaM + US, GA@CaMP + US). b) 2D reconstructed micro‐CT images in the defect sites at 8 weeks after implantation. c) Bone volume/total volume (BV/TV), d) trabecular thickness (Tb.Th), and e) trabecular number (Tb.N) results from micro‐CT (Control, GA@CaMP only, GA@MP + US, GA@CaM + US, GA@CaMP + US) (*n* = 3). f) H&E and g) MST staining of regenerated bone tissue in the defect sites (B: bone, Gel: residual gel, NB: newly formed bone tissue, MB: mineralized bone). h) Immunofluorescence staining of RUNX2 and i) Col‐1 in the defect area. j) Immunohistochemical staining of HIF‐1α in the defect area. Data are presented as Mean ± SD. Significance between multiple groups was calculated using one‐way ANOVA and Tukey–Kramer multiple comparisons test. ****p < 0.0001, ***p < 0.001, **p < 0.01, *p < 0.05, ns: no significance.

To evaluate the effectiveness of GA@CaMP for bone regeneration and repair under low‐frequency US, a cranial defect model was established (Figure S32, Supporting Information). The treatment flowchart is shown in **Figure** **7**a. After 8 weeks of treatment, micro‐CT imaging was used to evaluate defect regeneration. New bone formation was observed in all five groups, with the GA@CaMP + US group showing the most extensive inward bone growth (Figure 7b). To further analyze bone regeneration, we evaluated common bone parameters. The bone volume/total volume (BV/TV), which directly reflects bone mass changes, showed that at 8 weeks, the GA@CaMP + US group had a BV/TV of 30.97 ± 6.05%, significantly higher than the Control group (8.07 ± 1.19%), GA@CaMP group (11.87 ± 1.72%), GA@MP + US group (17.67 ± 2.51%), and GA@CaM + US group (19.30 ± 3.30%), indicating that the treatment accelerated new bone formation (Figure 7c). Additionally, the trabecular thickness (Tb.Th) of the treated groups was greater than that of the control group, with values of 0.23 ± 0.03, 0.37 ± 0.06, 0.42 ± 0.05, 0.42 ± 0.02, and 0.57 ± 0.05, respectively, demonstrating the effectiveness of GA@CaMP hydrogel in promoting bone mass (Figure 7d). We also evaluated the trabecular bone number (Tb.N), which reflects the degree of trabecular sparsity. The results showed that the Tb.N in the GA@CaMP + US group was 0.63 ± 0.03, which was significantly higher than that in the Control group (0.36 ± 0.10), GA@CaMP group (0.32± 0.04), GA@MP + US group (0.36 ± 0.03), and GA@CaM + US group (0.46 ± 0.07) (Figure 7e). This indicates that the GA@CaMP + US treatment can effectively support osteoblast‐mediated reconstruction of the trabecular network, resulting in a relatively denser bone structure, whereas bone repair in the other treatment groups mainly relied on trabecular thickening rather than the formation of new trabeculae.

H&E staining of the new bone at the defect site was conducted through tissue section images. H&E staining showed new bone formation in the control group, but most of the defect area was composed of loose fibrous tissue bridging the host bone edges (Figure 7f). In contrast, the treatment groups showed varying degrees of dense connective tissue formation covering the defect, with a larger number of osteoblasts and fibroblasts recruited and migrating into the area. This was particularly evident in the GA@CaM + US and GA@CaMP + US groups, where the residual hydrogel was surrounded and intertwined by newly formed osteoid‐like tissue, with fibrous tissue extending from both ends of the defect toward the center, indicating stronger bone regeneration potential. In the GA@CaMP + US group, new bone was observed to cover the defect in a wall‐like manner from both ends, occupying the largest area and exhibiting the best bone repair, consistent with the micro‐CT results. Notably, some tissue cells and osteoid‐like material were observed infiltrating the residual hydrogel, with no significant infection or tissue necrosis near the implantation site, confirming the biocompatibility and tissue integration capability of the hydrogel platform. Masson's staining, used to assess bone tissue maturity, revealed a high collagen fiber content in the newly formed osteoid, which stained blue, whereas fully calcified bone appeared red. Blue‐stained new bone was seen in the Control group, indicating the self‐repair capability of the bone, but the osteoid material was predominant without complete calcification (Figure 7g). In contrast, the treatment groups showed a gradual reduction in collagen content, with most showing calcification, and the osteoid was gradually replaced by red, mature bone, with the appearance and increase of bone lacunae, which are markers of mature bone. These changes accelerated the bone repair process. RUNX2, a key osteogenic transcription factor, is an important downstream signal in the classic Wnt signaling pathway that regulates osteoblast differentiation and bone formation.^[^ ^41^ ^]^ Upregulation of the RUNX2 gene expression indicates stronger osteogenic differentiation potential. After 8 weeks of hydrogel implantation, immunofluorescence staining was used to assess RUNX2 protein expression. The expression of osteogenic markers was significantly higher in the treatment groups, with the GA@CaMP + US group showing the strongest positive staining (Figure 7h and Figure S33a, Supporting Information). Col‐1 is a major component of the bone matrix, and its expression level directly reflects bone tissue synthesis. Immunofluorescence results showed that the expression of the Col‐1 marker was elevated to varying degrees in the treatment groups, with the highest levels observed in the GA@CaM + US and GA@CaMP + US groups (Figure 7i and Figure S33b, Supporting Information). These results suggest that the GA@CaMP hydrogel, under US stimulation, effectively promotes matrix maturation and mineralization during bone repair and remodeling. We further evaluated the oxygen‐generating capacity of the hydrogel platform by assessing the restoration of bone tissue oxygenation using immunohistochemical staining for HIF‐1α. The results showed that the Control group and the GA@MP + US group exhibited the largest brown‐stained areas, indicating the highest level of hypoxia (Figure 7j and Figure S33c, Supporting Information). In contrast, the other three groups effectively alleviated hypoxia by releasing dissolved oxygen from CaO_2_, leading to a significant downregulation of HIF‐1α expression. Meanwhile, we also observed that the GA@CaMP + US group exhibited significantly superior performance in promoting osteogenic differentiation and new bone formation compared to the GA@MP + US group lacking CaO_2_, confirming the sensitizing effect of supplemental oxygen within the bone repair strategy.

In summary, the constructed sono‐controllable Janus hydrogel platform presents an effective therapeutic strategy for alleviating local hypoxia and enhancing the expression of osteogenesis‐related proteins, thereby accelerating tissue repair and regeneration. This approach holds great promise for the treatment of bone defects caused by metastatic tumor invasion and post‐surgical procedures.

## 3 Conclusion

Bone metastasis is one of the most common and life‐threatening complications of advanced breast cancer, significantly impacting patient survival and quality of life. Traditional treatments primarily focus on tumor therapy, often neglecting the need for bone tissue repair. As a result, patients face an increased risk of bone loss, severe pain, and pathological fractures. Thus, the dual challenge of tumor treatment and bone regeneration remains a critical issue in breast cancer bone metastasis therapy. Advancements in nanotechnology have introduced novel nanomaterial‐based strategies for treating bone metastases in breast cancer. In recent years, researchers have designed multifunctional nanocarrier systems that combine the delivery of anticancer drugs with bone regenerative factors, aiming to achieve dual therapeutic effects of tumor treatment and bone repair.^[^ ^42^, ^43^ ^]^ Although some nanomaterials have shown promise in targeting tumor cells and promoting bone tissue repair, several challenges remain, including issues related to the long‐term biocompatibility of nanoparticles, achieving precise drug release, insufficient therapeutic efficacy, and the potential for side effects. Therefore, the ability to simultaneously achieve both functions within a single system remains a critical focus of ongoing research in this field.

This study introduces an innovative sono‐controllable Janus hydrogel platform, GA@CaMP, which integrates precise US‐mediated ROS release control, ZBP1 gene regulation, and the improvement of the hypoxic microenvironment, to address both tumor treatment and bone regeneration in breast cancer bone metastasis. A key innovation of our research is the dual role of ROS. Traditionally, ROS studies have focused primarily on their singular impact on either tumor treatment or tissue repair. Although the osteogenic effects of low‐concentration ROS have been recognized, the potential to leverage high‐concentration ROS for tumor cell eradication while simultaneously utilizing low‐concentration ROS for bone repair remains largely unexplored. We have achieved fine‐tuned, bidirectional regulation of ROS using US stimulation with temporal and spatial control, delivering dual therapeutic benefits of tumor elimination and bone regeneration within a single platform. Moreover, our study highlights the dual functionality of ZBP1 as a critical gene target. By upregulating ZBP1 expression, we not only enhanced ROS‐mediated tumor cell apoptosis but also stimulated the osteogenic differentiation of BMSCs, thereby facilitating bone repair. The injectability and controlled degradation of the hydrogel material ensure cell‐specific gene upregulation and sustained therapeutic effects. Here, it should be noted that the fundamental reason why ZBP1 triggers different downstream effects in the two cell models lies in the differences in their cellular microenvironments, particularly the varying levels of oxidative stress and ROS generation. ZBP1 is an innate immune sensor that recognizes Z‐form nucleic acids, particularly Z‐RNA, through its Zα domain and can activate programmed cell death pathways such as necroptosis. Its functional activation depends not on its expression level but on the extent of Z‐RNA accumulation.^[^ ^44^ ^]^ Under normal conditions, host cells rely on ADAR1‐mediated RNA editing to prevent aberrant accumulation of endogenous Z‐RNA and avoid misrecognition by ZBP1. However, when the ADAR1 function is impaired or Z‐RNA is produced excessively, ZBP1 can be aberrantly activated.^[^ ^45^ ^]^ Previous studies have shown that oxidative stress is a key inducer of Z‐RNA formation in a dose‐ and time‐dependent manner.^[^ ^36^ ^]^ In this study, high‐frequency US was used to induce substantial ROS generation in 4T1 cells, activating ZBP1 and promoting MLKL phosphorylation to trigger necroptosis. In contrast, low‐frequency ultrasound in 4T1 cells produced only low levels of ROS, insufficient to induce necroptosis, and the same was observed in BMSCs. Moreover, the unique stem cell microenvironment and osteogenic signaling pathways in BMSCs may redirect ZBP1 toward regulating proliferation and differentiation via pathways such as Wnt/β‐catenin. This characteristic further ensures the dual therapeutic effect.

The incorporation of CaO_2_ nanoparticles further alleviates tumor hypoxia, creating a favorable microenvironment for the synergistic effects of ROS modulation and ZBP1 regulation, thereby enhancing both tumor therapy and bone regeneration. However, it should be acknowledged that although the 7‐day oxygen release window of this system basically covers the key period of SDT treatment, effectively meeting the short‐term oxygen demand and enhancing the therapeutic effect, it only provides oxygen supply during the early stage compared to the longer‐term bone tissue regeneration process. Despite the relatively short duration of oxygen release, its beneficial effects on bone repair are still evident, mainly due to two factors: first, the timely oxygen supply during the initial stage of bone regeneration promotes angiogenesis and the recruitment and differentiation of osteoprogenitor cells; second, the byproduct Ca(OH)_2_ generated after oxygen release continuously releases Ca^2^⁺ and creates a mildly alkaline microenvironment, which further enhances osteogenic activity, facilitates bone matrix deposition, and activates multiple osteogenesis‐related signaling pathways. These combined effects effectively extend its bioactive window, achieving a synergistic strategy of “early‐stage oxygen supply plus sustained osteogenic stimulation.” In the future, we will further optimize the system to better match the dynamic and long‐term requirements of bone tissue regeneration.

In summary, our sono‐controllable Janus hydrogel platform integrates strategies of ROS modulation, ZBP1 regulation, and oxygen supplementation, offering a groundbreaking therapeutic approach for treating metastatic cancer and bone defects with clinically promising dual‐targeted effects. This study offers novel insights into the bidirectional regulation of ROS and the dual functionality of ZBP1, marking a major advancement in the development of multifunctional biomaterials for cancer therapy and bone regeneration.

## 4 Experimental Section

### Ethical Issues

All animal studies adhere to the Regional Ethics Committee for Animal Experiments' guidelines and received approval from the Laboratory Animal Center at Shanghai Tenth People's Hospital (Approval Number: SHDSYY‐2024‐Y3832).

### Materials and Reagents

Sigma–Aldrich provided all the chemicals for this study, except where noted otherwise. Ethanol was procured from Merck. The ZBP1 plasmid (Gene ID: VB231126‐1349kts and VB250223‐1129ffh) was purchased from GeneCopoeia Co., Ltd. GelMA (EFL‐GM‐60) and AlgMA (EFL‐AlgMA‐50K) were provided by Suzhou Intelligent Manufacturing Research Institute (Suzhou, China). The anti‐ZBP1 antibody was sourced from Thermo Fisher, while the anti‐pMLKL, anti‐RUNX2, and anti‐OPN antibodies were obtained from Affinity. The anti‐MLKL antibody was purchased from Abcam.

### Characterization

Transmission electron microscopy (TEM) and elemental mapping images were captured with a JEOL JEM 2100F field‐emission transmission electron microscope from Japan, set to an acceleration voltage of 200 kV. A scanning electron microscope (JSM 7800F, Japan) was used to examine the surface morphology and microstructure. Using the Malvern Zeta Particle Sizer Nano Series (Nano ZS90), particle size and zeta potential were assessed. The ultrasound device (Chattanooga 2776) was purchased from Shanghai Taijiu Medical Equipment Trading Co., Ltd. The generation of singlet oxygen (^1^O_2_) was monitored using 2,2,6,6‐tetramethyl‐4‐piperidone hydrochloride (TEMP). The concentration of dissolved O_2_ was measured using a dissolved O_2_ meter (Rex, JPBJ‐608). A universal testing machine (Instron 2344, USA) was used to assess the compressive strength of the hydrogel, applying a compression rate of 10 mm min^−1^ and reaching a maximum strain of 90% during the tests.

### Synthesis of pDNA@HMME‐ZIF8 (MHP)

At room temperature, 25 µL of an aqueous solution containing 25 µg of plasmid DNA (pDNA, DNase‐free) was slowly added to 125 µL of a 2‐methylimidazole aqueous solution containing 23.75 mg under mechanical stirring. Meanwhile, 10 µL of HMME (2 mg mL^−1^) was mixed with 125 µL of a Zn(NO_3_)_2_·6H_2_O solution (2.4 mg). The mixtures were stirred to ensure uniform blending. After 10 min, by adding the zinc nitrate complex solution drop by drop to the 2‐methylimidazole complex solution, a turbid solution was produced. After stirring and centrifugation at 10 000 rpm, a light pink precipitate, identified as MHP, was obtained. The product was washed three times and freeze‐dried for further use.

### Synthesis of CaO_2_ NPs

Under US treatment, 0.1 g of Calcium Chloride (CaCl_2_) and 0.35 g of polyvinylpyrrolidone (PVP) were fully dissolved in 15 mL of anhydrous ethanol. Subsequently, 1 mL of Ammonia Solution (NH_4_OH) was added under continuous stirring. After 30 min, 200 µL of Hydrogen Peroxide (H_2_O_2_) solution was added to the mixture, resulting in a pale blue milky solution. The product was collected after centrifugation and washing, and then freeze‐dried for future use.

### Ag arose Gel Electrophoresis Test

To prepare various loading samples, 4 µL of different aqueous nanomaterial solutions were combined with 1 µL of 5× DNA loading buffer. These samples were separately loaded into a 1% (w/v) agarose gel. The gel was run at 110 V for 40 min, and imaging was performed using a Gel Doc system under ultraviolet illumination.

### Evaluation of ^1^O_2_ Generation In Vitro

The ability of MHP to generate ^1^O_2_ under US irradiation (0.0, 0.3, 1.0, and 1.5 W cm^−2^) was characterized using the ^1^O_2_ indicator, 1,3‐diphenylisobenzofuran (DPBF). Briefly, DPBF solution was added to MHP solution (100 µg mL^−1^). The mixture was then irradiated with US at different intensities (1.0 MHz, 50% duty cycle) for a specified duration. After irradiation, the absorbance of the residual DPBF characteristic UV–visible absorption spectrum was measured using a UV–vis spectrophotometer.

### pH‐Responsive Release of pDNA from MHP Nanostructures

The release of pDNA from MHP nanoparticles in phosphate‐buffered saline (PBS) at pH levels of 5.0 and 7.2 was observed under stirring conditions. Supernatants were collected at different time points under various incubation conditions. The cumulative release of pDNA was evaluated by a UV–vis spectrophotometer.

### Cell Culture

Three types of cells were used in the in vitro experiments. Mouse‐derived breast cancer cells (4T1) and mouse bone marrow‐derived mesenchymal stem cells (BMSCs) were obtained from the Chinese Academy of Sciences. Luc+ 4T1 cells were sourced from OBIOTECH Co., Ltd (Shanghai). Both 4T1 and BMSCs were cultured in DMEM complete medium. Luc+ 4T1 cells were cultured in RPMI‐1640 complete medium.

### Detection of Intracellular ROS

4T1 cells were divided into control group, US group, MH group, MHP group, MP + US group, and MHP + US group. In brief, the 4T1 cells were exposed to the respective samples (40 µg mL^−1^) for 6 h, after which the medium was substituted with 2′,7′‐dichlorodihydrofluorescein diacetate (DCFH‐DA) for 30 min. The US treatment groups were subjected to US irradiation (1.5 W cm^−2^, 1.0 MHz, 50% duty cycle, 5 min). Intracellular ROS generation was marked by green fluorescence as seen through a fluorescence microscope.

### Evaluate the Cellular Internalization and Escape Abilities of MHP Nanoparticles

4T1 and BMSCs were pre‐seeded in CLSM‐specific culture dishes for 24 h. Subsequently, CLSM was used to observe the endocytosis situation of cells co‐incubated with FITC‐labeled MHP nanocarriers (40 µg mL^−1^) at different time points (0, 1, 3, 6 h). For lysosomal escape analysis, the cells were coincubated with FITC‐labeled MHP nanocarriers (40 µg mL^−1^) for a period of time, followed by US irradiation (4T1 cells: 1.5 W cm^−2^, 1.0 MHz, 50% duty cycle, 5 min; BMSCs cells: 0.3 W cm^−2^, 1.0 MHz, 50% duty cycle, 15 min) or without US for 3 h. The nuclei were stained blue with DAPI, and the lysosomes were stained red with LysoTracker. The lysosomal escape of the materials was then observed under CLSM.

### Evaluation of Cell Viability

To evaluate the in vitro cytotoxicity of MHP composite nanoparticles, 4T1 cells and BMSCs were coincubated with MHP nanocarriers at different concentrations (0, 10, 20, 30, 40, and 50 µg mL^−1^) for 24 h in 96‐well plates. Using the standard CCK‐8 assay, cell viability was measured. To assess the cytotoxic effects on 4T1 cells under different treatments, 4T1 cells were divided into control group, US group, MH group, MHP group, MH + US group, and MHP + US group. The concentrations of MH and MHP were both 40 µg mL^−1^. After adding them and co‐incubating for 6 h, specific groups were subjected to US (1.5 W cm^−2^, 1.0 MHz, 50% duty cycle, 5 min). The in vitro therapeutic effects were evaluated using the standard CCK‐8 assay.

### Preparation of GelMA/AlgMA Hybrid Hydrogel (GA Hydrogel)

The hydrogel was named X:Y GA hydrogel, where X:Y represents the mass ratio between GelMA and AlgMA, with the detailed mass ratio provided in TableS1 (Supporting Information). Briefly, GelMA and AlgMA were weighed separately and dissolved at a concentration of 10% w/v in double‐distilled water (ddH_2_O) containing lithium acylphosphate (LAP) salt (0.3% w/v) to generate precursor solutions A and B. Different volumes of precursor solutions A and B were mixed according to the desired mass ratio, followed by thorough stirring to form a composite solution. The final sterile polymer solution underwent filtration using a 0.22 µm membrane and was poured into specific molds. The gelation was achieved by ultraviolet (405 nm) photopolymerization.

### Preparation of Injectable GA@CaMP

Based on the cytotoxicity evaluation of BMSCs, an appropriate amount of MHP was thoroughly dissolved in precursor solution B, while an appropriate amount of CaO_2_ was dissolved in precursor solution A. The two solutions were mixed at a mass ratio of GelMA:AlgMA = 7:3. The final composite solution was stirred until uniform, ensuring that the MHP concentration reached 200 µg mL^−1^ and the CaO_2_ concentration reached 600 µg mL^−1^. This resulted in the GA@CaMP polymer solution. The polymer solution was then placed into specific molds and photopolymerized under ultraviolet light (405 nm) for 5–10 s to form a gel. The composition of the hydrogel can be adjusted according to the previously established method to generate hydrogels with different compositions.

### Cell Morphology Observation

To observe the morphology of cells seeded on GA hydrogel, a 4% paraformaldehyde solution was used to fix BMSCs. After permeabilization with 0.1% Triton, the cells were stained with phalloidin‐TRITC and DAPI, with F‐actin appearing green and the cell nuclei appearing blue.

### Characterization of Hybrid Hydrogel

The sol–gel transition of the hydrogel was analyzed using the inverted bottle method, and the gelation time was recorded. At room temperature, the compression curve of the mixed hydrogel, after reaching its equilibrium swelling, was recorded on an Instron 2344 microtester (compression speed: 10 mm min^−1^, compression strain: 90%). To evaluate the swelling properties of the hydrogel, the hydrogel was freeze‐dried at −80 °C to obtain a dried hydrogel, and its weight (mdry) was recorded. The hydrogel was then immersed in PBS (pH 7.4) and removed at 0, 4, 8, 12, 24, 36, and 48 h. The surface water of the hydrogel was blotted dry, and the weight of the swollen hydrogel (mwet) was recorded. The hydrogel's swelling ratio was determined using this formula:

(1) $$\mathrm{The}\;\mathrm{swelling}\;\mathrm{rate}\;\left(\%\right)=\left(m\;\left(\mathrm{wet}\right)-m\left(\mathrm{dry}\right)\right)/m\left(\mathrm{dry}\right)\times 100\%$$

To explore the hydrogel's degradation process, the initial weight of the freeze‐dried hydrogel sample (W₀) was recorded. The hydrogel was then immersed in PBS containing 2 U mL^−1^ type II collagenase. At predetermined time points, the remaining hydrogel samples were weighed to determine their dry weight (W₁). The degradation rate of the hydrogel was calculated as follows:

(2) $$\mathrm{The}\;\mathrm{degradation}\;\mathrm{rate}\;\left(\%\right)=\left(W_{0}-W_{1}\right)/W_{0}\times 100\%$$

### Detection of O_2_ Production in GA@CaMP

The oxygen release profile of the hydrogel was obtained under hypoxic conditions. Specifically, GA@CaMP was immersed in nitrogen‐deoxygenated PBS containing 100 U mL^−1^ catalase, and then placed in a hypoxic incubator. The oxygen release was monitored daily for 10 consecutive days using a dissolved oxygen meter. The degree of cellular hypoxia and intracellular oxygen levels were measured using a hypoxia probe—the oxygen‐sensitive indicator [Ru(dpp)_3_]Cl_2_. Normoxic 4T1 and BMSC cells were seeded in 6‐well plates at an appropriate density, and hypoxia was induced using a cell‐specific anaerobic culture pouch for 24 h. The GA@CaMP hydrogel was then added to the hypoxic environment for co‐incubation with the cells for 48 h. Subsequently, the medium was replaced with 100 µL of [Ru(dpp)_3_]Cl_2_ solution (500 µg mL^−1^ in DMSO). After 30 min of incubation, the cells were thoroughly washed with fresh PBS, and fluorescent images were captured using a fluorescence microscope.

### Assessment of Osteogenic Potential In Vitro

A transwell assay was conducted to simulate a co‐culture environment. The cells were split into four groups at random: G1 (CON), G2 (GA@CaMP), G3 (GA@CaM + US), and G4 (GA@CaMP + US). Specifically, BMSCs were placed in the lower chamber of a transwell insert. The hydrogel groups had the hydrogel placed in the upper chamber (100 µL), while the CON group had PBS in the upper chamber as a replacement. After 24 h, the medium in all groups was replaced with osteogenic induction medium (DMEM complete medium with the addition of 50 µg mL^−1^ L‐ascorbic acid, 10 mM β‐glycerophosphate disodium salt, and 10 nM dexamethasone) and changed every three days. Different treatments were applied, with US treatment (0.3 W cm^−2^, 1.0 MHz, 50% duty cycle, 15 min) applied to the designated groups. On day 7 of co‐culture, the cells were fixed and then stained with 1× BCIP/NBT staining solution. To determine alkaline phosphatase (ALP) activity, 100 µL of Western and IP cell lysis buffer (without inhibitors) was added. After centrifugation, the supernatant was collected, and ALP activity was measured by the BCA kit. On day 14 of co‐culture, Alizarin Red S (ARS) staining was performed. The procedure was similar to the ALP staining. After the cells were fixed and washed, 2% ARS staining solution was added. Thirty minutes later, the mineralized matrix was observed under an optical microscope. Following this, each well received a 10% solution of cetylpyridinium chloride. After 15 min, the absorbance of the supernatant at 562 nm was measured to quantify the amount of mineralized matrix. To evaluate the gene transcription levels of typical osteogenic markers, RT‐qPCR analysis was performed on day 7 of co‐culture. The mRNA expression levels of the cells were normalized to the housekeeping gene GAPDH, and data were calculated using the 2‐ΔΔCT method. The primer sequences used in this study are listed in Table S2 (Supporting Information).

### Neutralizing ROS

Dissolve the n‐acetyl‐l‐cysteine powder in PBS solution and add it to the sample at a concentration of 0.0625×10^−3^ m, and carry out the US procedure.

### In Vivo Safety Evaluation

Healthy BALB/c mice were divided into the PBS group, the GA@CaMP group, and the GA@CaMP + US group. The hydrogel was injected subcutaneously into the mice's dorsal region, with a volume of 100 µL per mouse. US treatment was applied to the implanted hydrogel area every other day (1.5 W cm^−2^, 1.0 MHz, 50% duty cycle, 5 min), for a total of four treatments. Mouse body weight was recorded every other day from Day 0 to Day 21. On Day 21, blood samples were collected for analysis of liver and kidney function markers, as well as blood biochemical parameters. Major organs were also collected for sectioning and staining to evaluate biological safety.

### Assessment of the Release of MHP In Vivo

MHP nanomaterials were labeled with the IR‐783 dye and encapsulated within the GA@CaMP hydrogel platform (200 µg mL^−1^). The hydrogel was subcutaneously set into the dorsal area of healthy BALB/c mice. At Days 0, 5, 10, 15, and 20, fluorescence intensity and spatial distribution changes on the mice's dorsal region were monitored using an in vivo imaging system (IVIS).

### Efficacy of Antitumor Activity In Vivo

Three different models were established to simulate various tumor treatment scenarios: the orthotopic breast cancer model, the postoperative tumor recurrence model, and the breast cancer bone metastasis model. The construction methods and efficacy evaluations for each model are as follows: Orthotopic breast cancer model: 4T1 cells (1×10^6^ per mouse) were injected into the fourth left mammary fat pad of BALB/c mice, and the mice were divided into six groups: (G1) CON group; (G2) CaMP + US (in solution form); (G3) GA@CaMP group; (G4) GA@MP + US group; (G5) GA@CaM + US group; (G6) GA@CaMP + US group. On Day 1, tumors were injected with the gel or solution (100 µL), and on Days 2, 4, 6, and 8, the mice were subjected to US treatment (1.5 W cm^−2^, 1.0 MHz, 50% duty cycle, 5 min). Tumor volume and body weight were recorded. The volume formula is as follows: Volume (V, mm^3^) = (Length × Width^2^)/2. On Day 14, the tumors of mice were collected for weighing, photography, and subsequent fixation in formaldehyde, embedding, and sectioning for various staining. Major organs were also collected for sectioning and staining to evaluate biological safety. Postoperative tumor recurrence model: Luc‐4T1 cells (1×10^6^ per mouse) were injected into the fourth left mammary fat pad of BALB/c mice. When the tumor volume reached 100 mm^3^, almost the entire tumor tissue was surgically excised. The mice were then randomly divided into five groups: (G1) CON group; (G2) GA@CaMP group; (G3) GA@MP + US group; (G4) GA@CaM + US group; (G5) GA@CaMP + US group. Starting from Day 1 post‐surgery, 100 µL of the gel was injected into the wound site, and a transparent, waterproof membrane dressing was applied to secure the gel in place. US treatment (1.5 W cm^−^^2^, 1.0 MHz, 50% duty cycle, 5 min) was applied on Days 2, 4, 6, and 8 for each group. Bioluminescence imaging was performed before surgery, and on Days 8, 12, and 16 to monitor tumor recurrence and quantify fluorescence. On Day 16, the recurrent tumors were excised, weighed, photographed, and subjected to CD3^+^CD4^+^ and CD3^+^CD8^+^ staining for immune evaluation. The survival rate of the mice was monitored from Day 0 to Day 40. Breast cancer bone metastasis model: each mouse received an injection of Luc‐4T1 cells (1×10^5^ per mouse) into the bone marrow cavity along the tibia's long axis. On Day 7 after injection, IVIS confirmed the formation of metastatic tumors in the tibia. The mice were then divided into three groups: (G1) CON group; (G2) GA@CaMP; (G3) GA@CaMP + US group. On Day 1, the gel (100 µL) was injected into the metastatic tumor site, and US treatment (1.5 W cm^−2^, 1.0 MHz, 50% duty cycle, 5 min) was applied on Days 2, 4, 6, and 8. From Days 0 to 14, the circumference of both lower legs was recorded every other day. IVIS was performed on Days 5, 10, and 14 to monitor the growth of metastatic tumors. On Day 14, the metastatic tumors were collected, weighed, and photographed. H&E staining and Micro‐CT (PerkinElmer, Quantum GX II, USA) scanning were performed on the tumors. Lung tissue was collected and stained to observe the metastatic lung nodules.

### In Vivo Bone Regeneration

The bone repair experiment was conducted using a rat cranial critical‐size defect model, with rats randomly assigned to five groups: (G1) CON group; (G2) GA@CaMP group; (G3) GA@MP + US group; (G4) GA@CaM + US group; (G5) GA@CaMP + US group. An incision was made on the scalp of the deeply anesthetized male Sprague Dawley rats. The tissue was bluntly separated until the cranial bone was fully exposed. The periosteum was carefully incised and protected. A circular critical‐size defect (5 mm) was created on the right side of the midline of the cranium. The sterilized gel platform was then implanted into the defect area, with the CON group using rats that did not receive any material as the control. The skin was then carefully sutured, and the rats were given intraperitoneal injections of cefuroxime antibiotic to prevent potential infection. In the US‐treated groups, the rats were subjected to US treatment on Days 1, 5, 10, and 14 after surgery (0.3 W cm^−2^, 1.0 MHz, 50% duty cycle, 15 min). After 8 weeks, the rats’ cranial bone was harvested. The defect area was analyzed for mineralization using micro‐computed tomography (micro‐CT, Bruker, Germany). The ZKKS‐MicroCT4.1 program was used to calculate related bone parameters. The cranial bone tissue was stained with H&E staining, Masson's trichrome, RUNX2, Col‐1 immunofluorescence staining and HIF‐1α immunohistochemical staining.

### RNA Sequencing

The breast cancer bone metastasis mice were divided into the control group (no treatment) and the treatment group (GA@CaMP + US). The treatment group received a tumor injection of the gel, followed by four sessions of US treatment as described for the breast cancer bone metastasis mice. Sequencing and analysis were then performed by Shanghai OE Biotechnology Co., Ltd.

### Statistical Analysis

All experimental data are presented as the mean ± standard deviation (Mean ± SD). The sample size (*n*) corresponding to each statistical analysis is specified in the figure legends of the relevant result graphs. In this study, the following statistical methods were used to evaluate the significance of differences between groups, with the significance level set at *α* = 0.05 (i.e., a *P*‐value < 0.05 was considered statistically significant). Details are as follows:​ For multiple group comparisons, one‐way analysis of variance (one‐way ANOVA) was applied. If the ANOVA test indicated an overall significant difference, post‐hoc tests (e.g., Tukey's method) were further used for pairwise comparisons between groups. Kaplan–Meier survival analysis was performed to assess animal survival status, and the log‐rank test was used to determine the significance of differences in survival between groups. The significance annotation rules are as follows: **** denotes *p* < 0.0001, *** denotes *p* < 0.001, ** denotes *p* < 0.01, * denotes *p* < 0.05, and “ns” indicates no statistical significance. All statistical analyses in this study were conducted using GraphPad Prism 9.5.1 software.

## Conflict of Interest

The authors declare no conflict of interest.

## Author Contributions

Y.L., Y.L., and C.L. contributed equally to this work. H.H.Y., H.X.X., and Y.F.Z. designed the research strategy and supervised the whole process. Y.T.L., Y.Y.L., Y.C.L., and X.J.C. synthesized and characterized the nanoparticles. Y.T.L., Y.Y.L., C.Y.L., X.J.C., Y.Z., H.S., J.Y.Q., S.Z., T.X.W., and X.W.W. conducted in vitro and in vivo experiments, collected and recorded data. Y.T.L., Y.Y.L., and Y.C.L. wrote the paper. All the authors contributed to the discussion of the results and implications, and edited the manuscript at all stages.

## Supporting information



Supporting Information

## Data Availability

The data that support the findings of this study are available from the corresponding author upon reasonable request.
