# Supplementary material for: Sono‐Controllable Janus Hydrogel Platform for Sequential Tumor Eradication and Bone Regeneration in Metastatic Breast Cancer
Source: Adv Sci (Weinh). 2025 Sep 4;12(43):e06386. doi: 10.1002/advs.202506386 (PMC12631841; doi:10.1002/advs.202506386)
Supplement: Supplementary file 1 — Supporting Information [file ADVS-12-e06386-s001.docx]

Supporting Information

**Sono-Controllable Janus Hydrogel Platform for Sequential Tumor Eradication and Bone Regeneration in Metastatic Breast Cancer**

*Yitong Li^1, 3#^, Yunyun Liu^2#^, Chuyun Lou^4#^, Xuejun Chen^5^, Ying Zhang^1, 3^, Hui Shi^1, 3^, Jiayi Qiu^1, 3^, Shen Zhang^1, 3^, Taixia Wang^1, 3^, Xiaowei Wang^6^, Haohao Yin^2*^, Huixiong Xu^2*^, Yifeng Zhang^7*^*


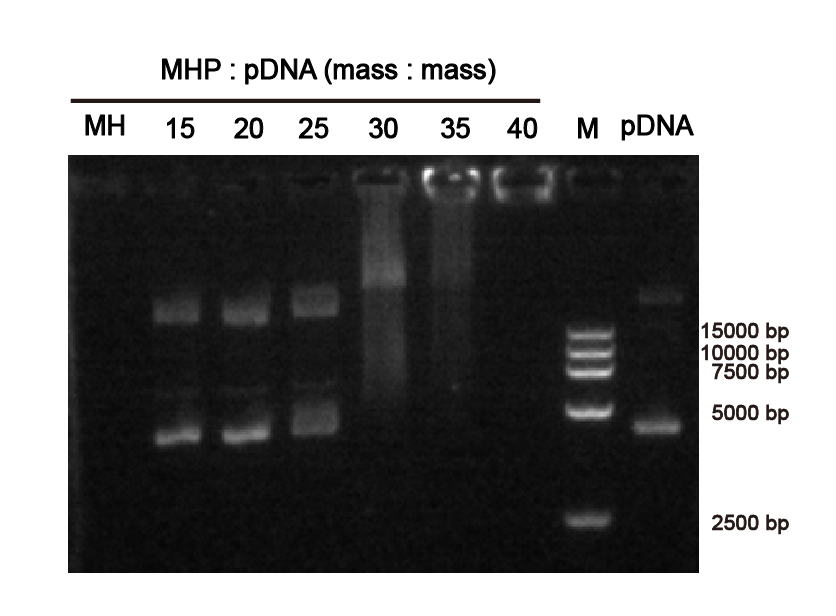


**Figure S1.** 1% agarose gel image. Lanes 1–7: MH, MHP/pDNA at mass ratios of 15 : 1, 20 : 1, 25 : 1, 30 : 1, 35 : 1, and 40 : 1, respectively; Lanes 8 and 9: DNA ladder and naked pDNA.


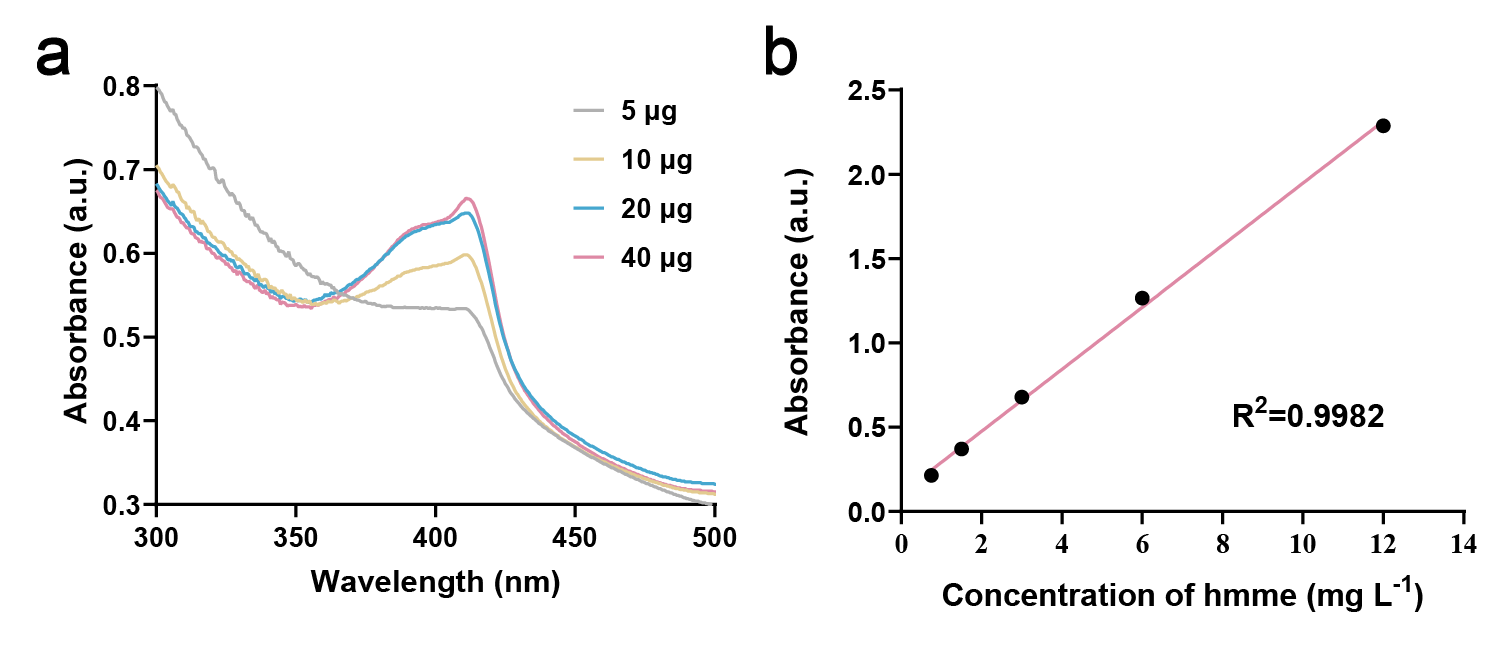


**Figure S2.** **a)** UV-vis spectra of MHP loaded with different amounts of HMME (5, 10, 20, and 40 µg). **b)** Standard curve for HMME.


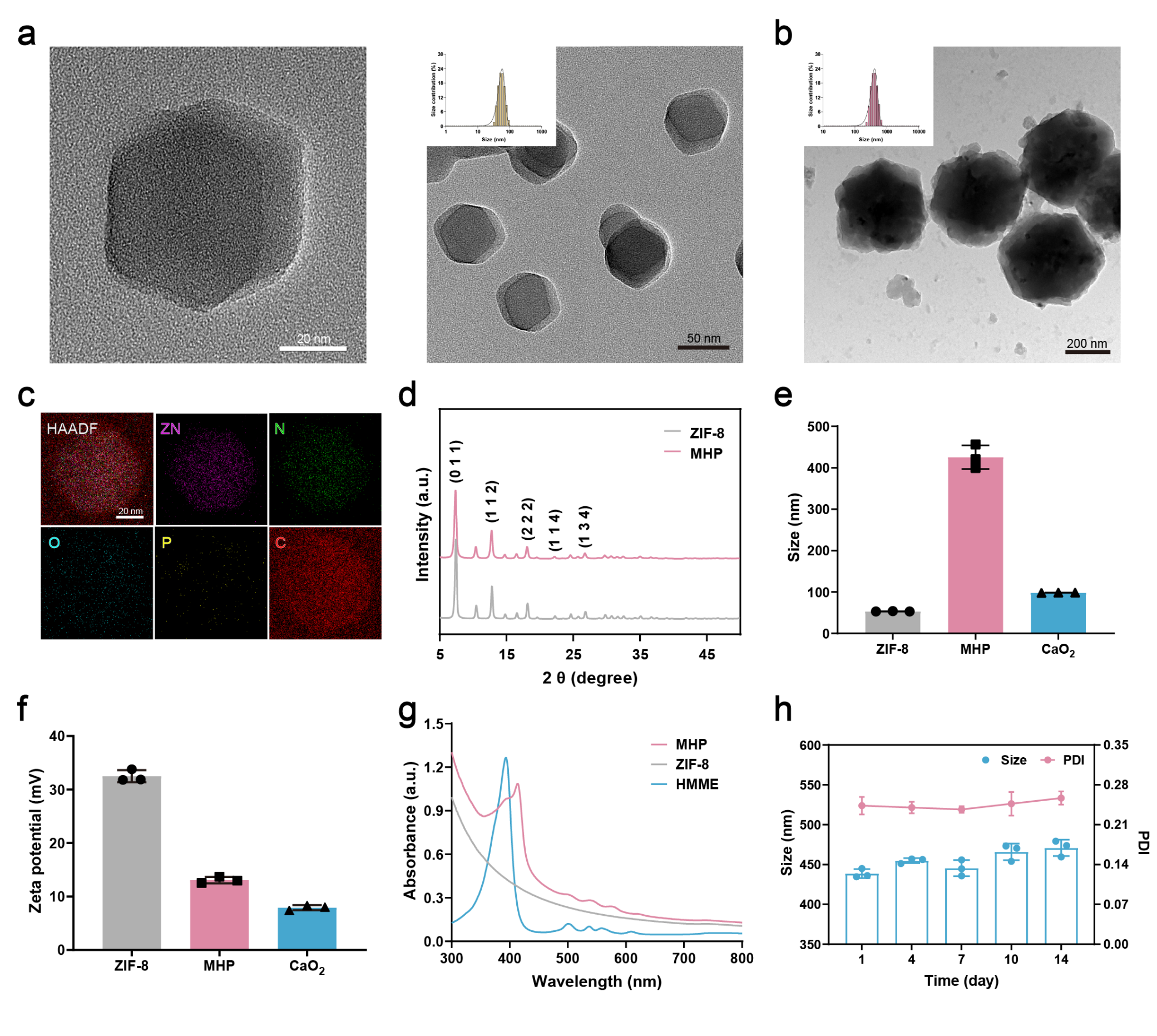


**Figure S3.** **a)** TEM images of ZIF-8. **b**) TEM image of MHP. **c)** Corresponding elemental mappings of ZIF-8. **d)** XRD analysis of ZIF-8 and MHP. **e)** Particle size and **f)** zeta potential of ZIF-8, MHP, and CaO₂ NPs (n = 3). **g)** UV-vis spectra of HMME, ZIF-8, and MHP. **h)** The change of hydrated particle size and polydispersity index (PDI) of MHP after storage in PBS (pH = 7.4) for different days (Day 1, 4, 7, 10, 14) (n = 3). Data are presented as Mean ± SD.

**
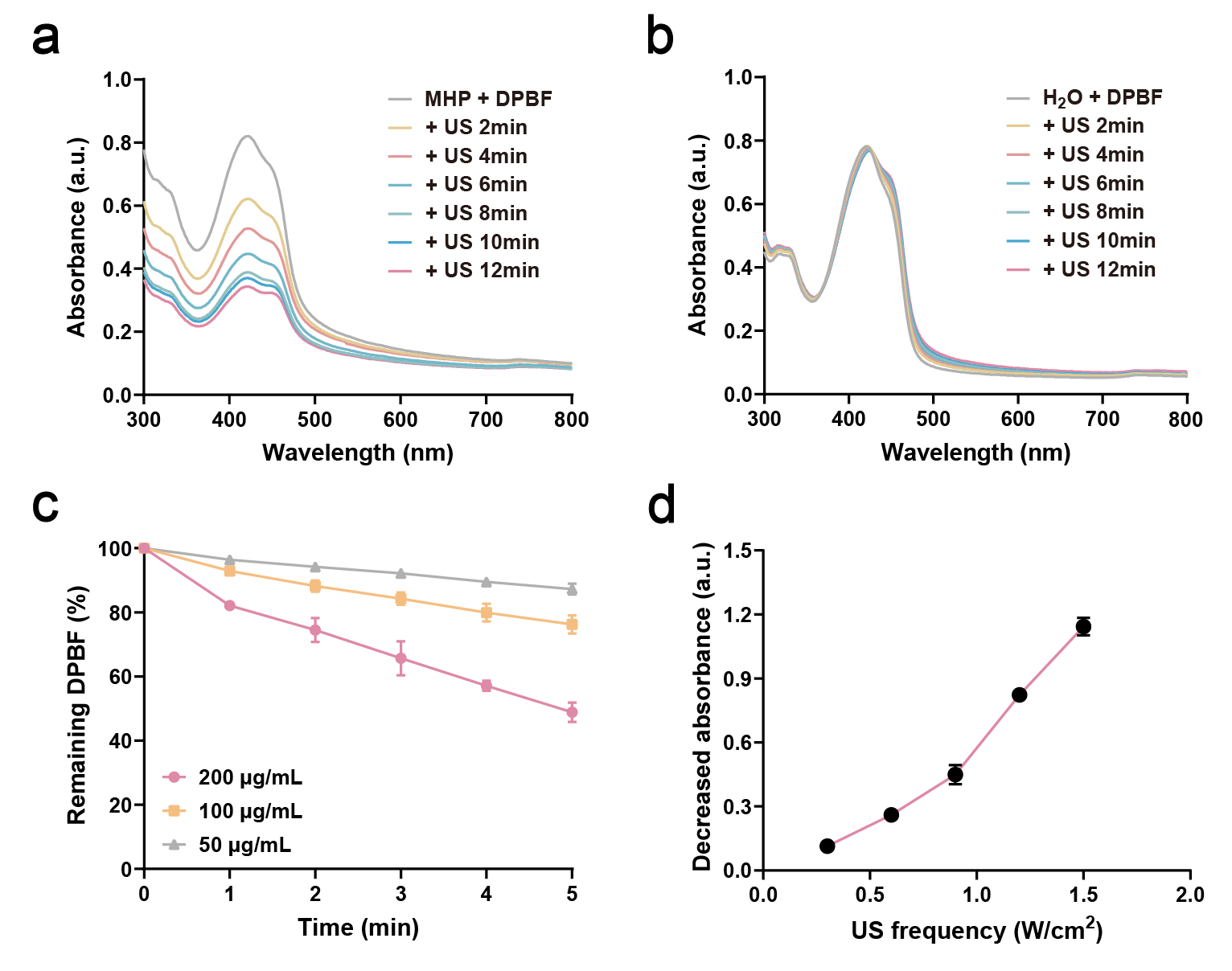
**

**Figure S4.** **a)** Under prolonged US irradiation, the UV-vis absorption spectra of DPBF with MHP. **b)** Absorption spectra of DPBF under prolonged US irradiation. **c)** Concentration-dependent (50, 100, and 200 μg/mL) absorbance changes of DPBF co-incubated with MHP under US irradiation (n = 3). **d)** Response curve showing the change in consumed DPBF with varying US frequencies (0.3, 0.6, 0.9, 1.2, 1.5 W/cm²) (n = 3). Data are presented as Mean ± SD.


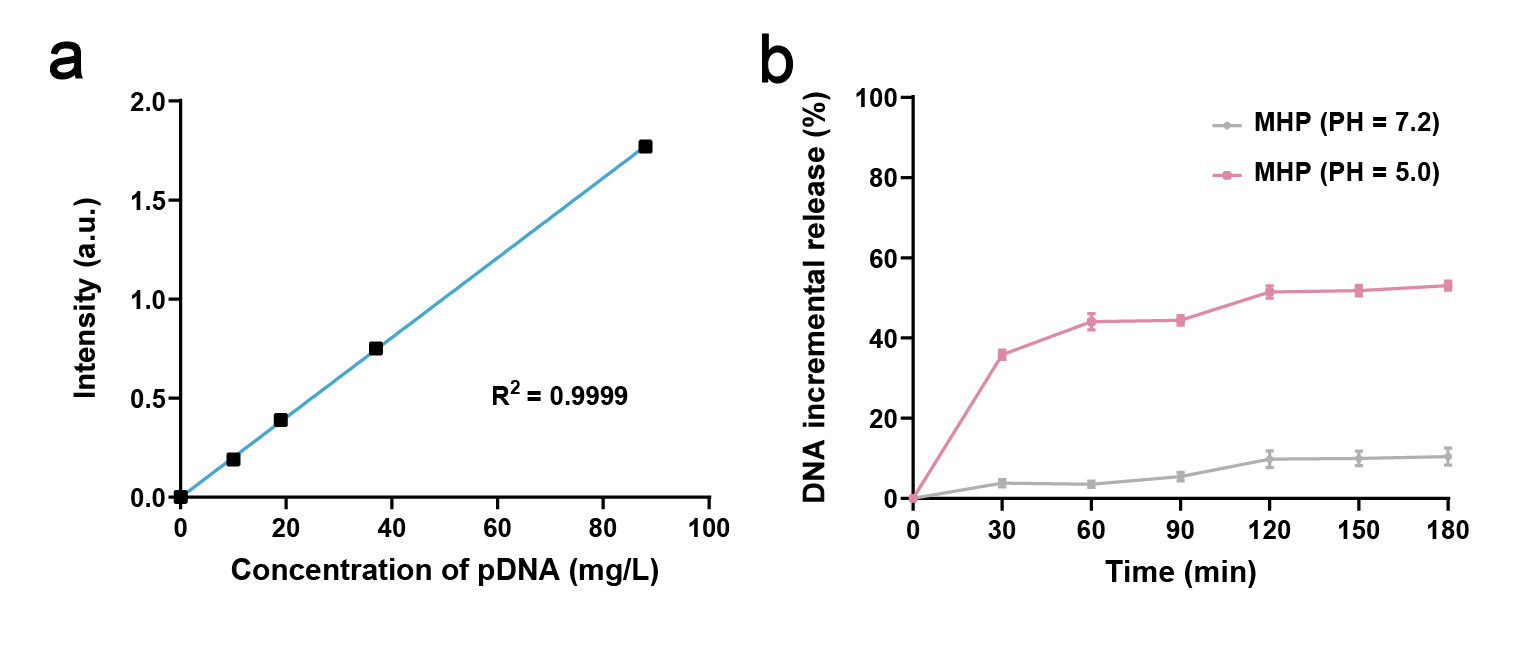


**Figure S5.** **a)** Standard curve for pDNA. **b)** The incremental release rate of pDNA from MHP in PBS at pH 7.2 and 5.0 (n = 3). Data are presented as Mean ± SD.

**
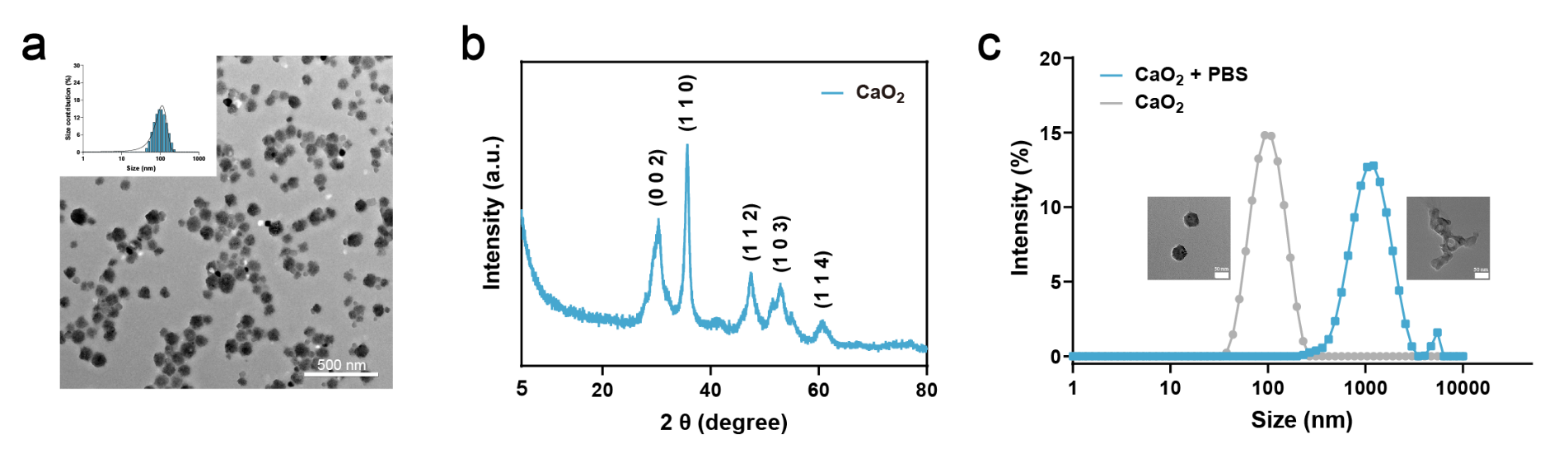
**

**Figure S6.** **a)** TEM image (scale bar = 500 nm) and **b)** XRD analysis of CaO₂ NPs. **c)** The change of particle size distribution of CaO₂ after storage in PBS (pH = 7.4) for 24h.


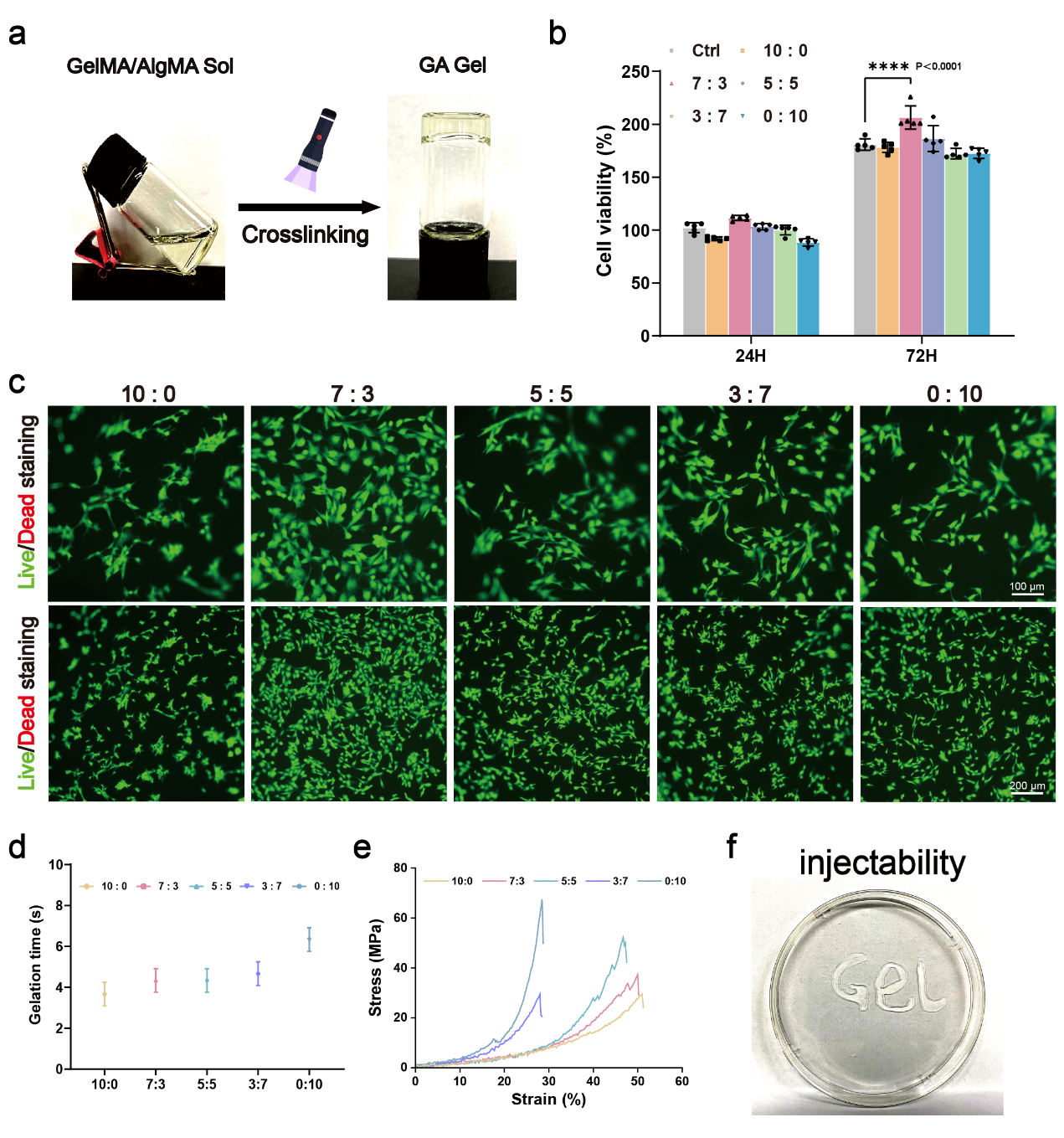


**Figure S7. a)** Photographs showing the formation of GA hydrogels. **b)** Cell viability of BMSCs seeded on different GA hydrogels (GelMA/AlgMA mass ratios of 10 : 0, 7 : 3, 5 : 5, 3 : 7, and 0 : 10) after 1 and 3 days of culture, with untreated cells serving as the blank control (n = 5). **c)** Live/dead staining of BMSCs seeded on GA hydrogels with different compositions (10 : 0, 7 : 3, 5 : 5, 3 : 7, and 0 : 10). **d)** Gel formation time (n = 3), and **e)** compressive stress-strain curves of different GA hydrogels, including 10 : 0 (yellow), 7 : 3 (pink), 5 : 5 (green), 3 : 7 (purple), and 0 : 10 (blue). **f)** Photographs showing the injectability of GA hydrogels. Data are presented as Mean ± SD. Significance between multiple groups was calculated using one-way ANOVA and Tukey-Kramer multiple comparisons test. ****P < 0.0001, ***P < 0.001, **P < 0.01, *P < 0.05, ns: no significance.


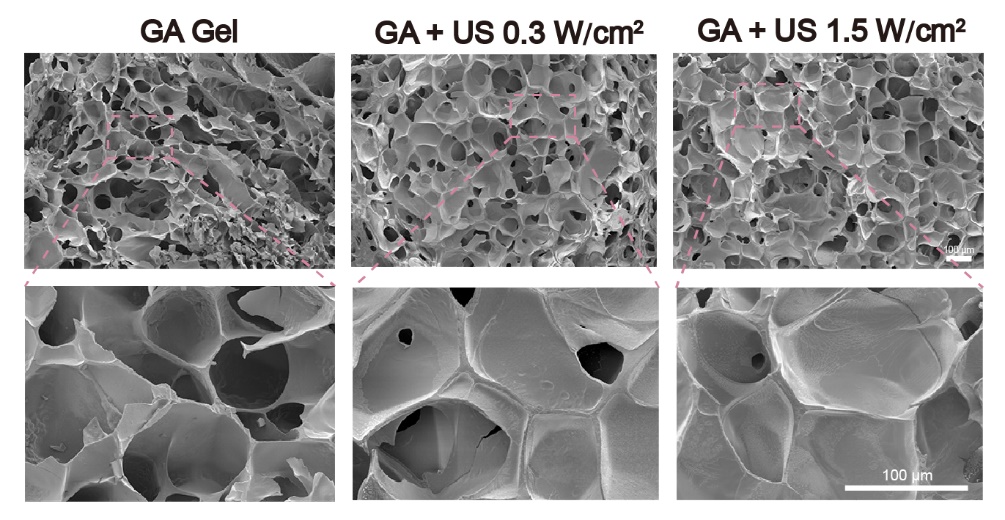


Figure S8. SEM images of GA hydrogels subjected to US irradiation at different frequencies (0, 0.3, and 1.5 W/cm^2^).


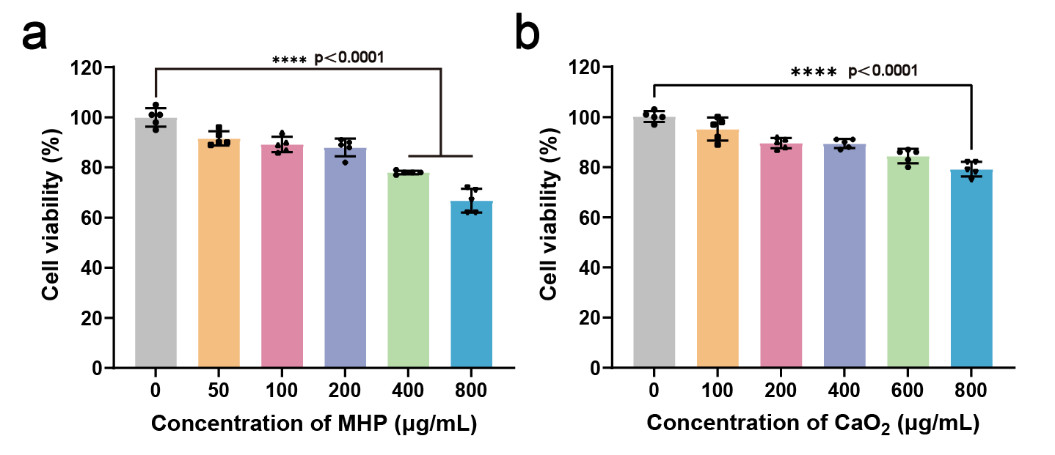


Figure S9. a) Cell viability of BMSCs co-cultured with different concentrations (0, 50, 100, 200, 400, and 800 µg/mL) of MHP encapsulated in GA hydrogels for 72 h (n = 5). b) Cell viability of BMSCs co-cultured with different concentrations (0, 100, 200, 400, 600, and 800 µg/mL) of CaO₂ encapsulated in GA@MP hydrogels for 72 h (n = 5). Data are presented as Mean ± SD. Significance between multiple groups was calculated using one-way ANOVA and Tukey-Kramer multiple comparisons test. ****P < 0.0001, ***P < 0.001, **P < 0.01, *P < 0.05, ns: no significance.


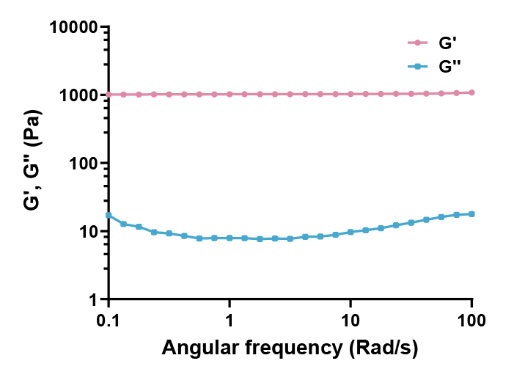


Figure S10. The elastic (G’) and viscous (G”) moduli of GA@CaMP hydrogels vary with frequency.


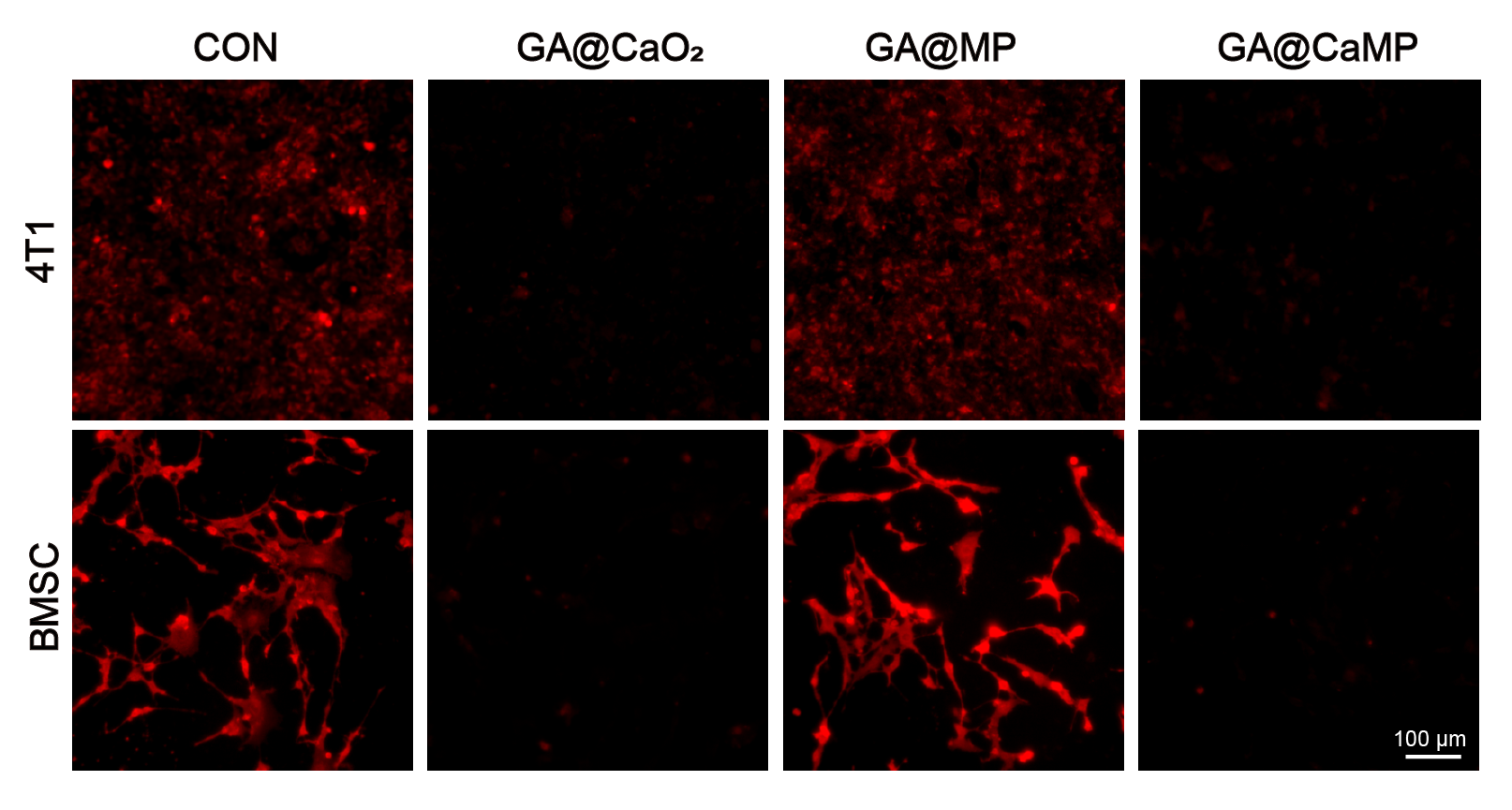


**Figure S11.** Fluorescence images of [Ru(dpp)_3_]Cl₂-stained 4T1 and BMSC cells after different treatments for 48h (Control, GA@CaO₂, GA@MP and GA@CaMP).


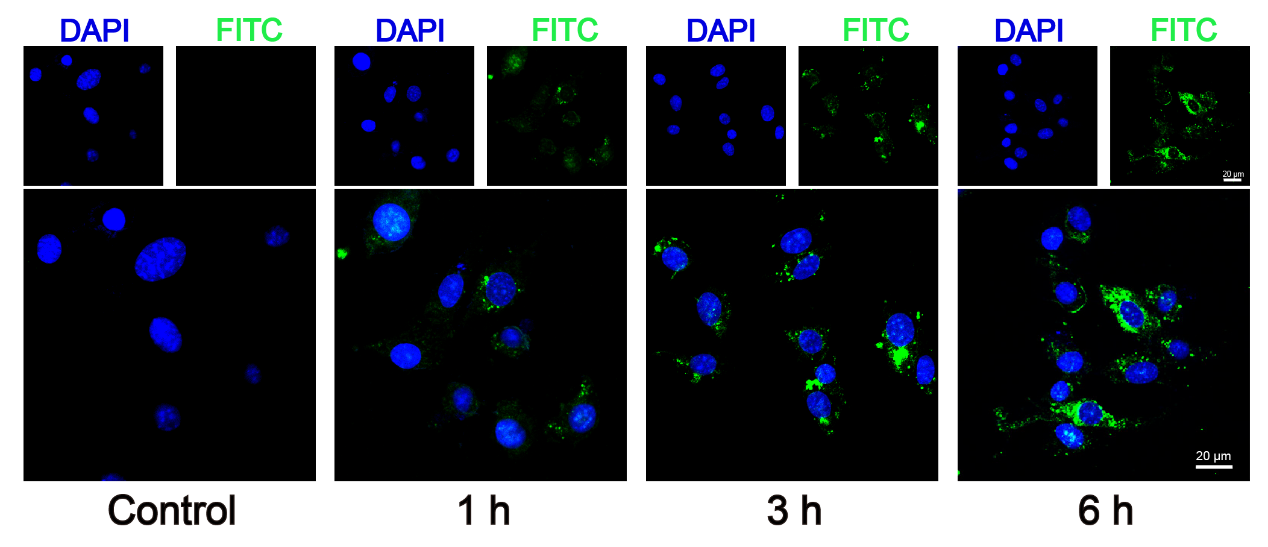


**Figure S12.** CLSM images of FITC-labeled MHP uptake by BMSCs at different time points (0, 1, 3, and 6 h).


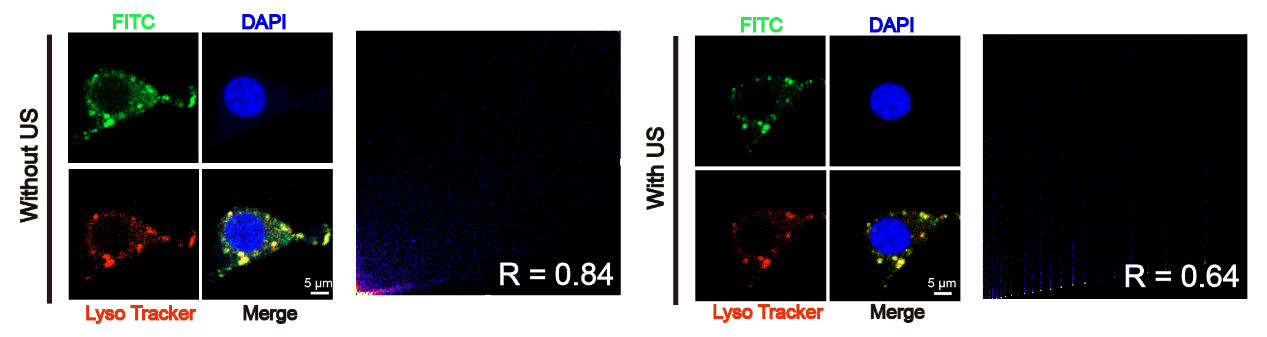


**Figure S13.** Representative CLSM images and corresponding confocal imaging study of BMSCs co-stained with LysoTracker Red and FITC-labeled MHP, with or without US. R represents the Pearson correlation coefficient.


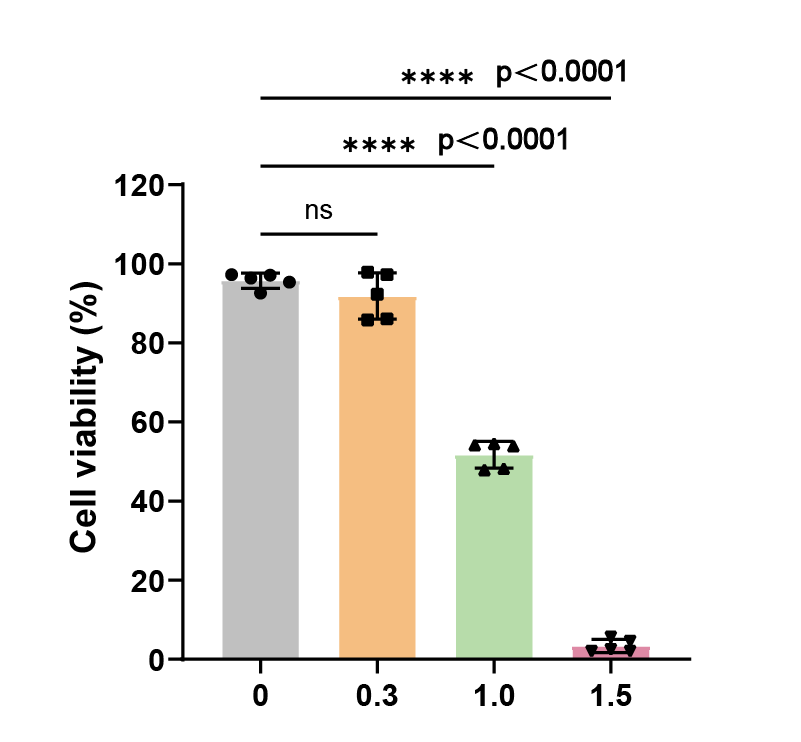


**Figure S14.** Antitumor efficacy of MHP with different US frequencies (0, 0.3, 1.0, and 1.5 W/cm^2^) under 5 min US irradiation (n = 5). Data are presented as Mean ± SD. Significance between multiple groups was calculated using one-way ANOVA and Tukey-Kramer multiple comparisons test. ****P < 0.0001, ***P < 0.001, **P < 0.01, *P < 0.05, ns: no significance.


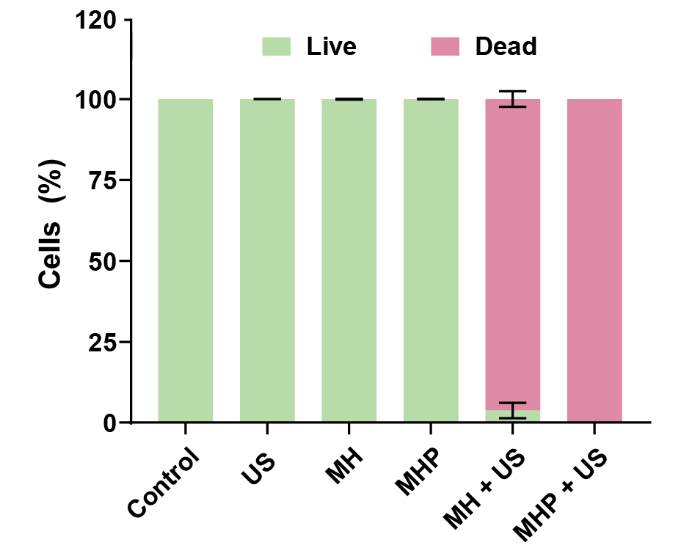


**Figure S15.** Quantification of the PI/calcein-AM fluorescence intensity ratio of 4T1 cells after different treatments, including Control, US only, MH, MHP, MH + US and MHP + US (n = 3). Data are presented as Mean ± SD.


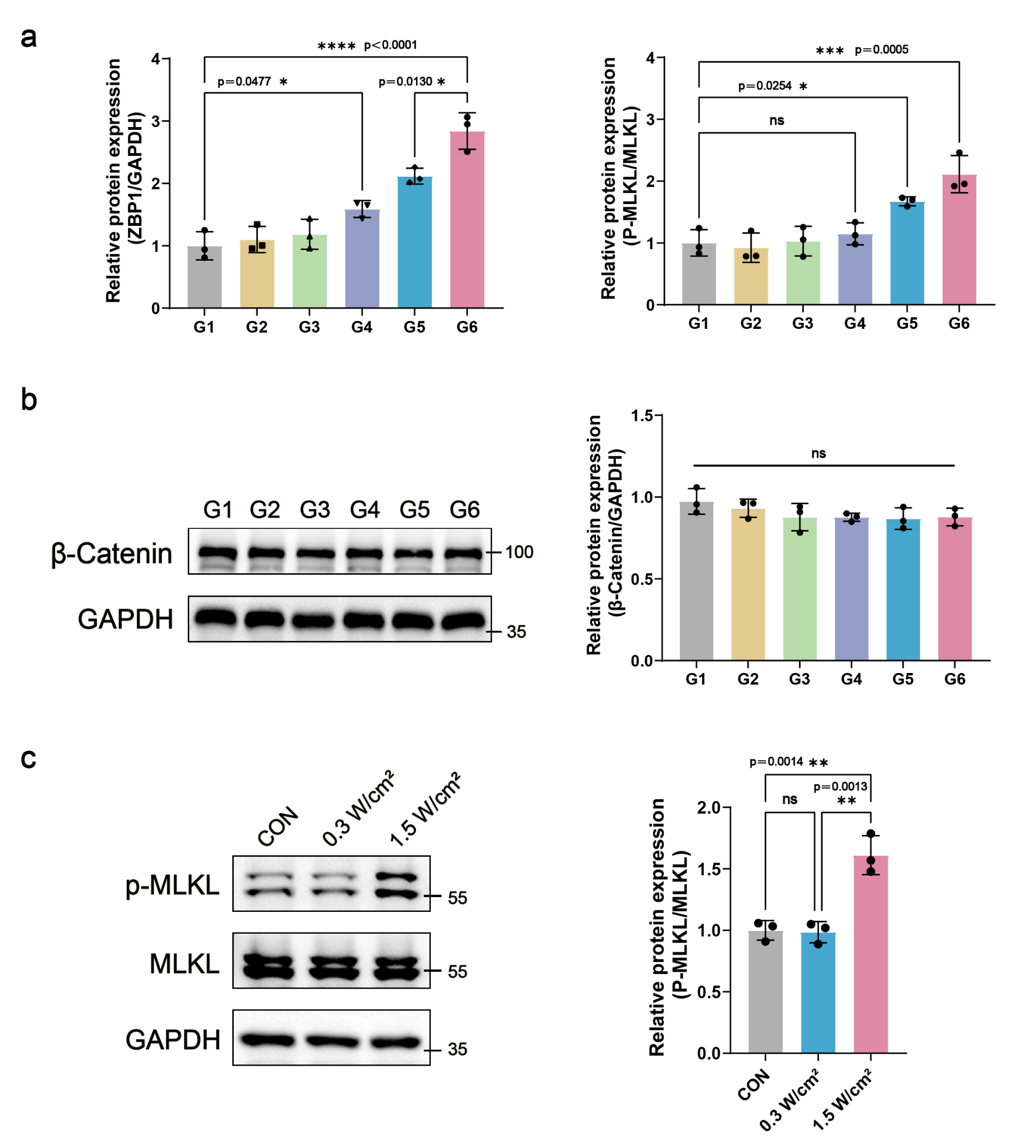


**Figure S16. a)** Related quantitative analysis of western blot analysis of ZBP1 and necroptosis-related proteins expression levels in 4T1 cells (G1: Control, G2: US only, G3: MH, G4: MHP, G5: MH + US, and G6: MHP + US) (n = 3). **b)** Western blotting analysis and related quantitative analysis of β-catenin protein expression level in 4T1 cells (G1: Control, G2: US only, G3: MH, G4: MHP, G5: MH + US, and G6: MHP + US) (n = 3). **c)** Western blotting analysis and related quantitative analysis of necroptosis-related proteins expression levels in 4T1 cells after different treatments, including CON, MHP + US (0.3 W/cm²), and MHP + US (1.5 W/cm²) (n = 3). Data are presented as Mean ± SD. Significance between multiple groups was calculated using one-way ANOVA and Tukey-Kramer multiple comparisons test. ****P < 0.0001, ***P < 0.001, **P < 0.01, *P < 0.05, ns: no significance.


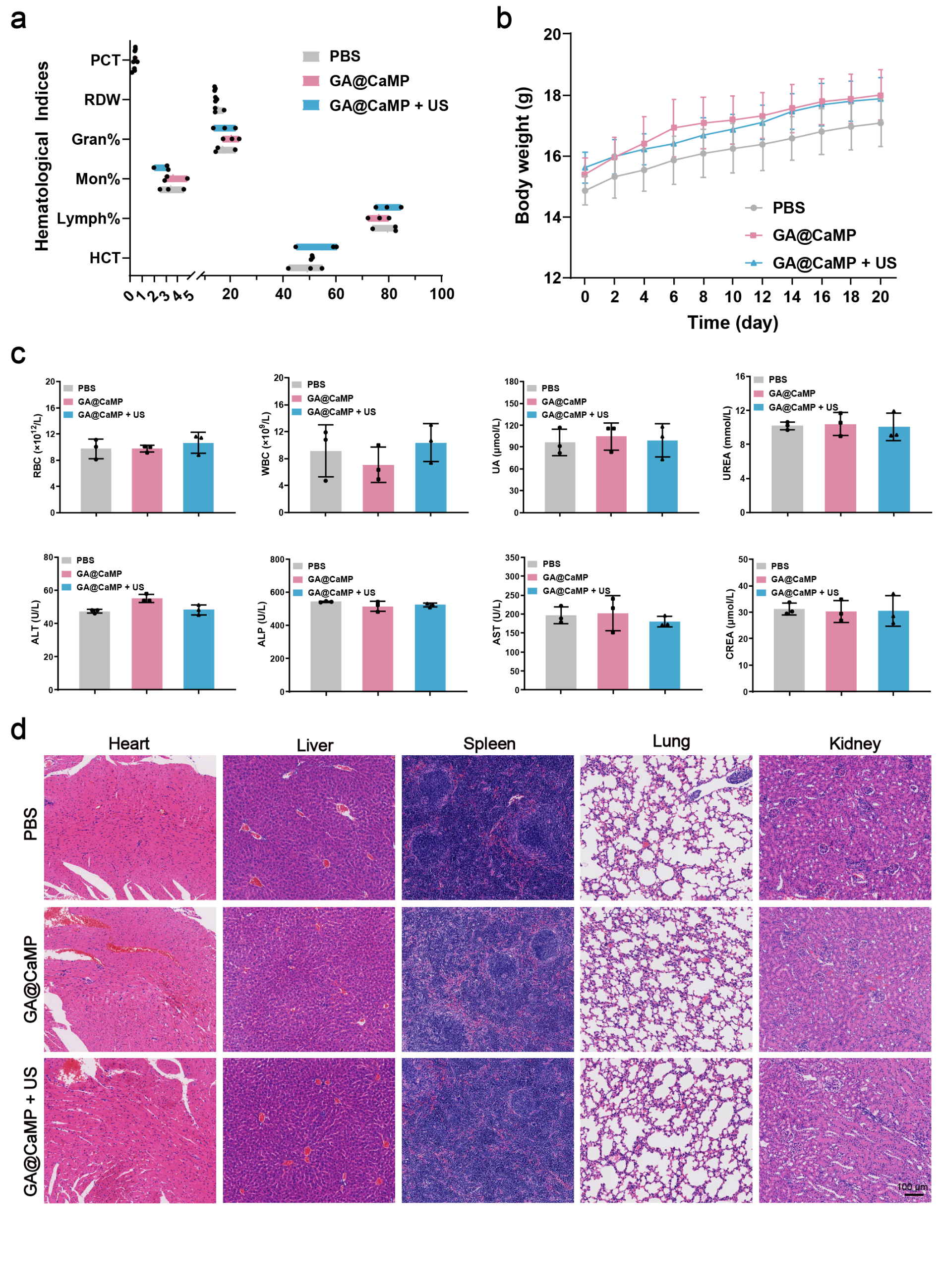


**Figure S17.** **a)** Hematology parameters of mice after different treatments, including PBS, GA@CaMP, GA@CaMP + US. Mice treated with PBS solution served as Control (n = 3). **b)** Changes of mice body weights in different groups (PBS, GA@CaMP, GA@CaMP + US) (n = 3). **c)** Hematology parameters, blood biochemical indexes, and **d)** H&E staining of the major organs of mice after different treatments, including PBS, GA@CaMP, GA@CaMP + US (n = 3). Data are presented as Mean ± SD.


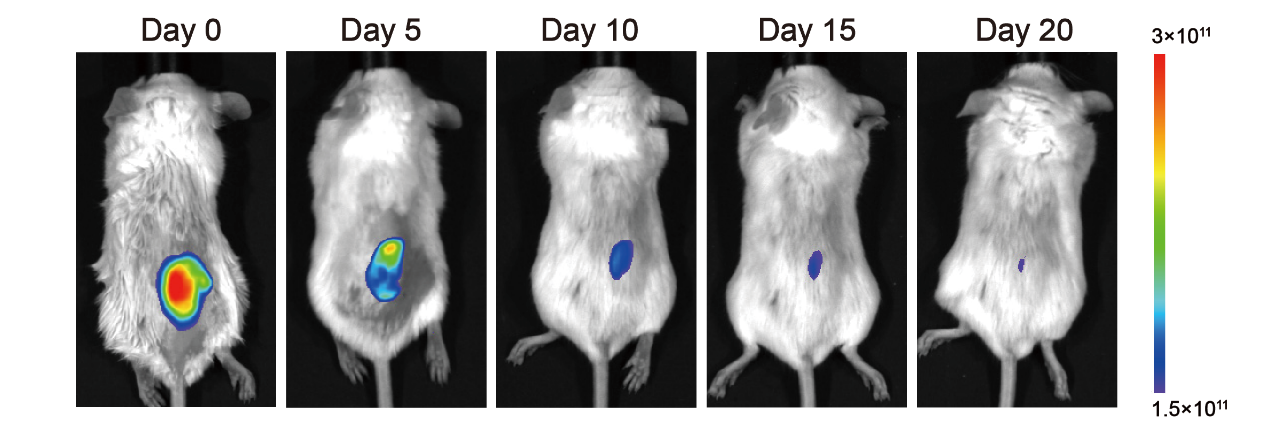


Figure S18. In vivo retention of IR783-labeled MHP in GA@CaMP gel for various days (Day 0, 5, 10, 15, and 20).


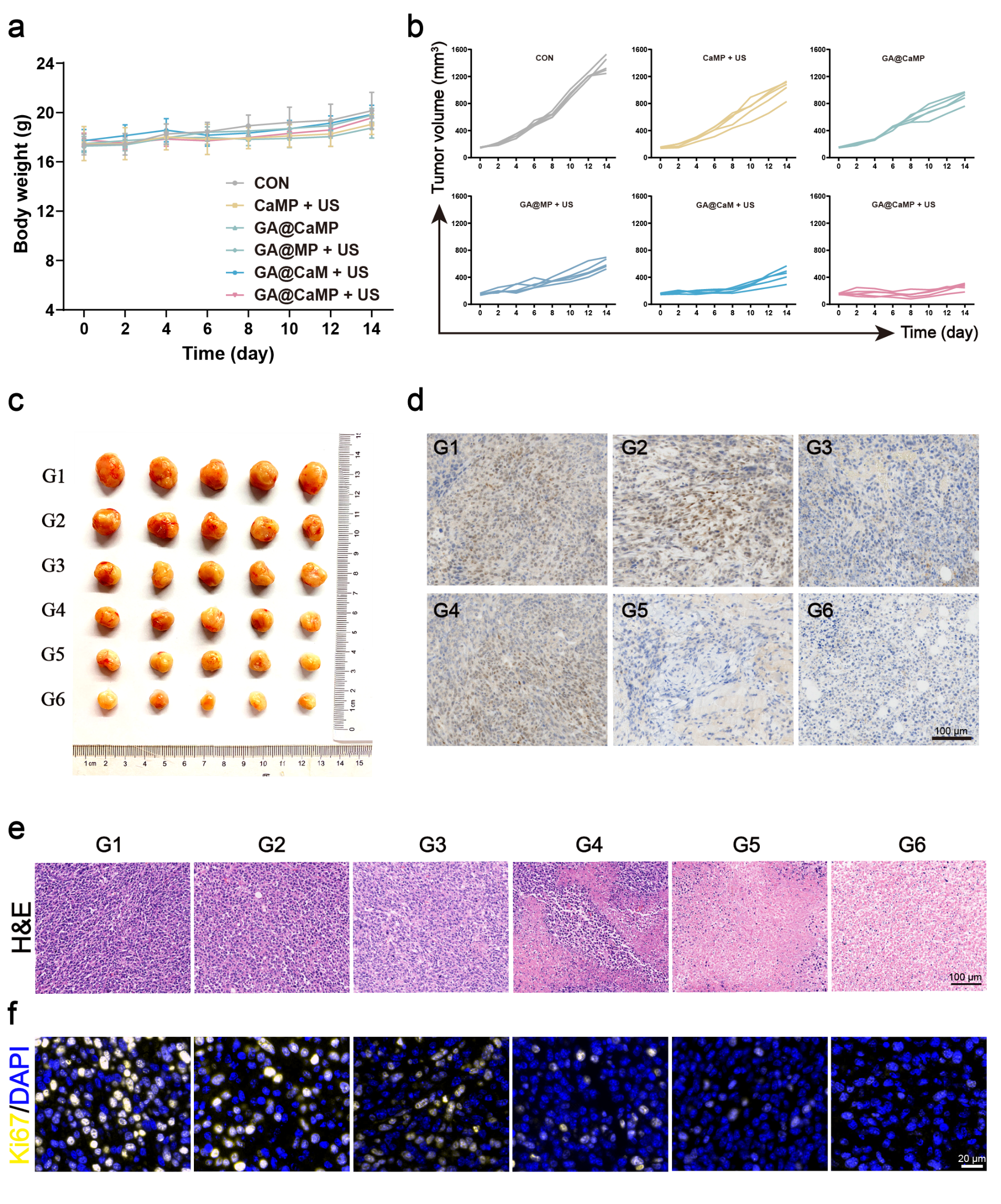


**Figure S19. a)** Time-dependent body weight curves of mice with different treatments, including Control, CaMP + US, GA@CaMP only, GA@MP + US, GA@CaM + US, GA@CaMP + US (n = 5). **b)** The progression of tumors in mice after undergoing various treatments including Control, CaMP + US, GA@CaMP only, GA@MP + US, GA@CaM + US, GA@CaMP + US (n = 5). **c)** Photos of the tumors collected from in situ tumor models after 14 days of different treatments (G1: Control, G2: CaMP + US, G3: GA@CaMP only, G4: GA@MP + US, G5: GA@CaM + US, G6: GA@CaMP + US) (n = 5). **d)** HIF-1α staining of tumor tissues after different treatments (G1: Control, G2: CaMP + US, G3: GA@CaMP only, G4: GA@MP + US, G5: GA@CaM + US, G6: GA@CaMP + US). **e)** H&E and **f)** Ki67 staining results of tumors from mice after different treatments (G1: Control, G2: CaMP + US, G3: GA@CaMP only, G4: GA@MP + US, G5: GA@CaM + US, G6: GA@CaMP + US). Data are presented as Mean ± SD.


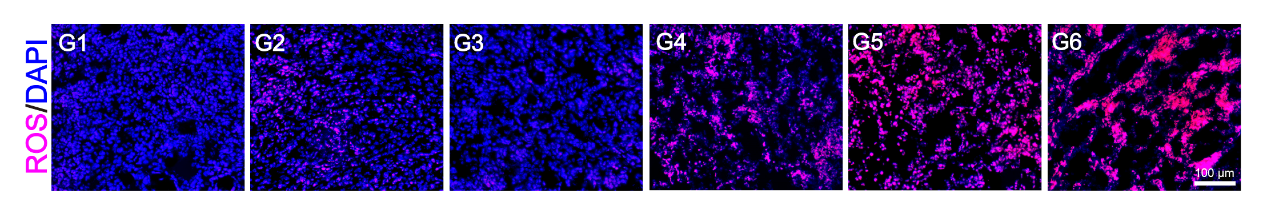


**Figure S20.** Representative ROS staining images of tumor tissues from mice after different treatments (G1: Control, G2: CaMP + US, G3: GA@CaMP only, G4: GA@MP + US, G5: GA@CaM + US, G6: GA@CaMP + US).


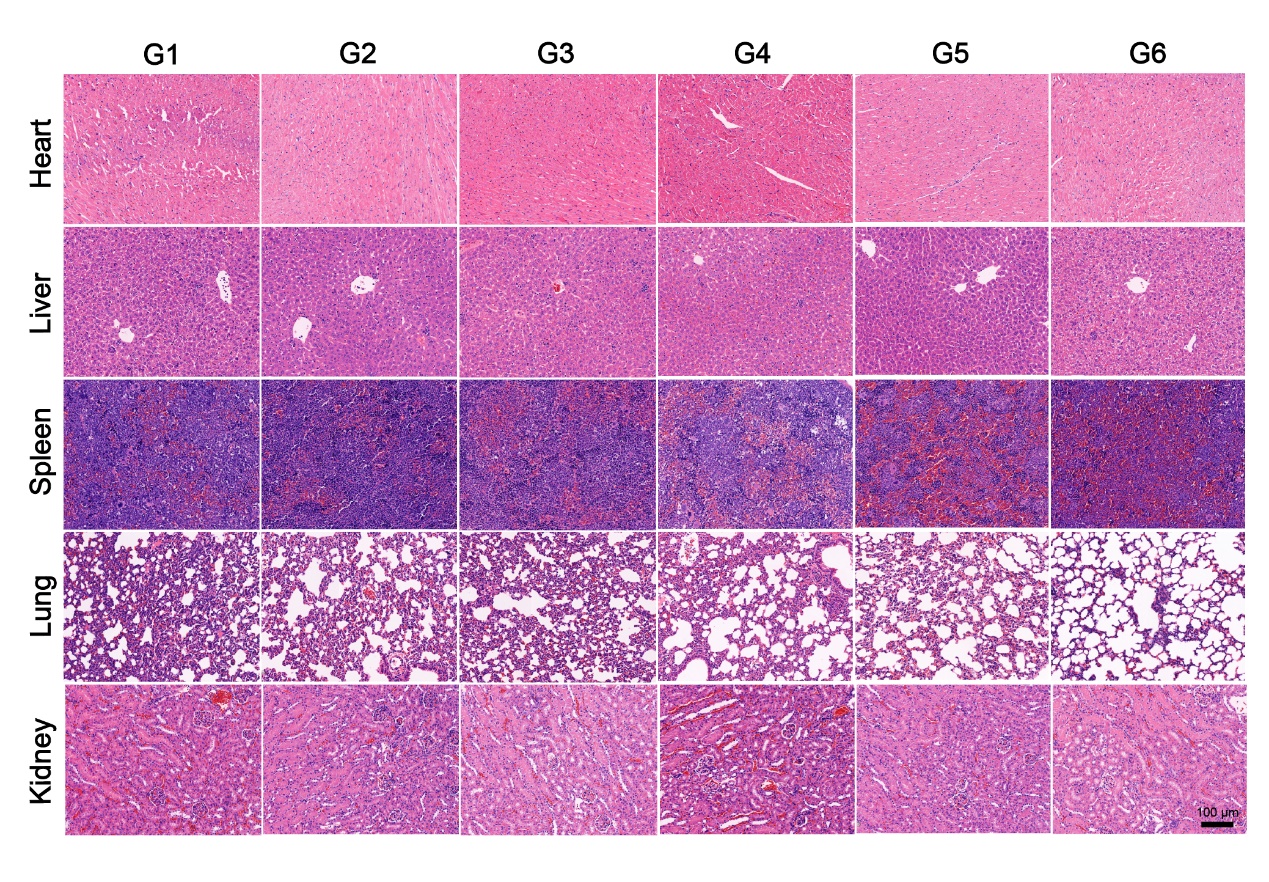


Figure S21. H&E staining of the major organs harvested from 4T1 tumor-bearing mice on the 14th day from different groups (G1: Control, G2: CaMP + US, G3: GA@CaMP only, G4: GA@MP + US, G5: GA@CaM + US, G6: GA@CaMP + US).


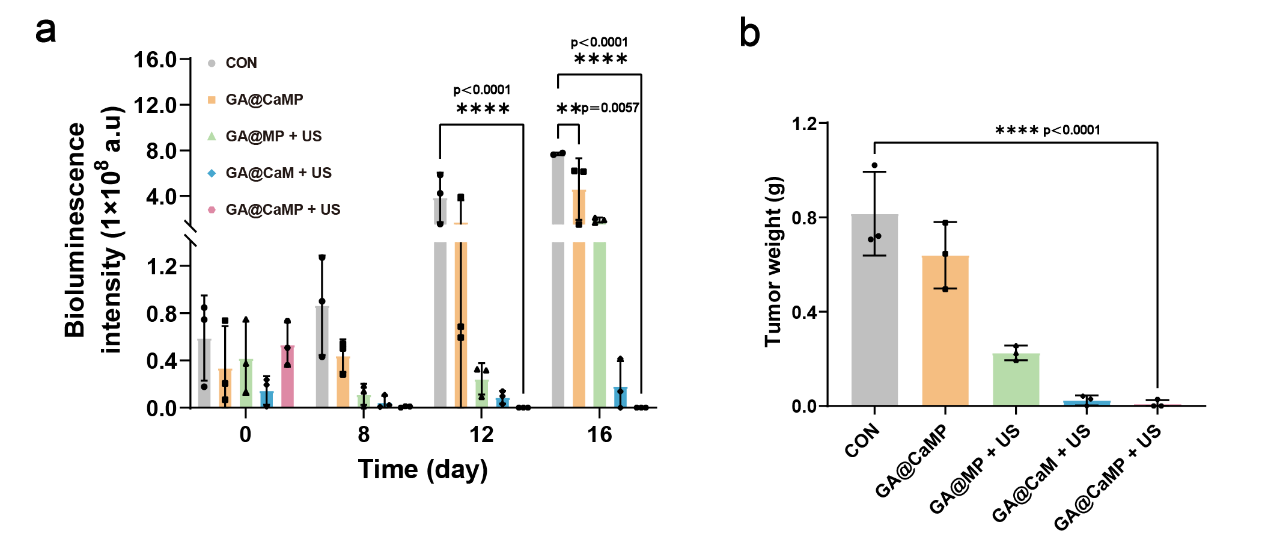


Figure S22. **a)** Fluorescence intensity of tumors measured at different time points (Day 0, Day 8, Day 12, and Day 16) in mice after various treatments, including Control, GA@CaMP only, GA@MP + US, GA@CaM + US, GA@CaMP + US (n = 3). **b)** Average weight of tumors excised from mice with residual tumors following different treatments, including Control, GA@CaMP only, GA@MP + US, GA@CaM + US, GA@CaMP + US (n = 3). Data are presented as Mean ± SD. Significance between multiple groups was calculated using one-way ANOVA and Tukey-Kramer multiple comparisons test. ****P < 0.0001, ***P < 0.001, **P < 0.01, *P < 0.05, ns: no significance.


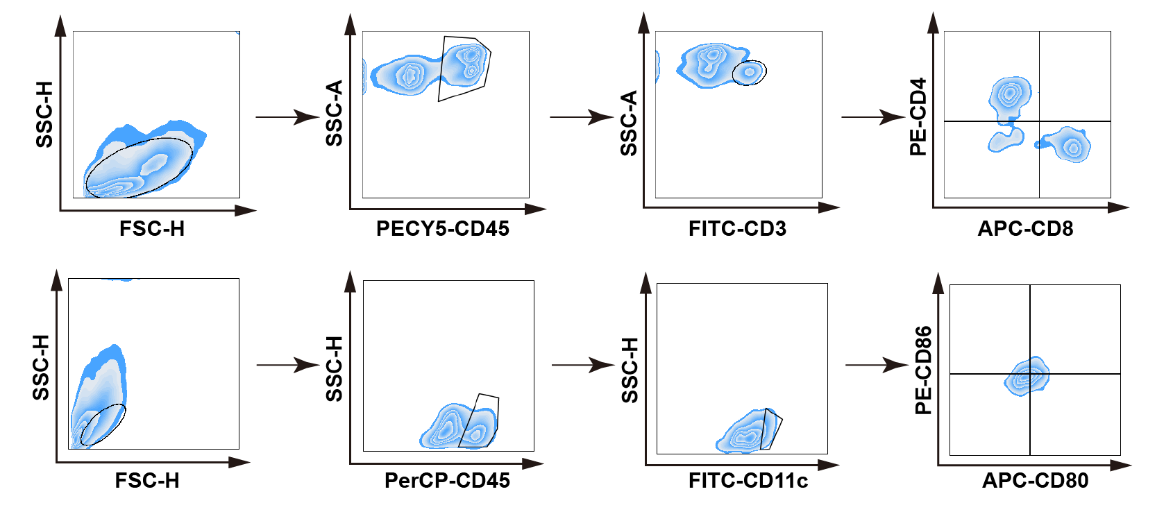


**Figure S23.** Gating strategies for isolating CD4^+^ and CD8^+^ T cells and mature DCs from tumor tissues.


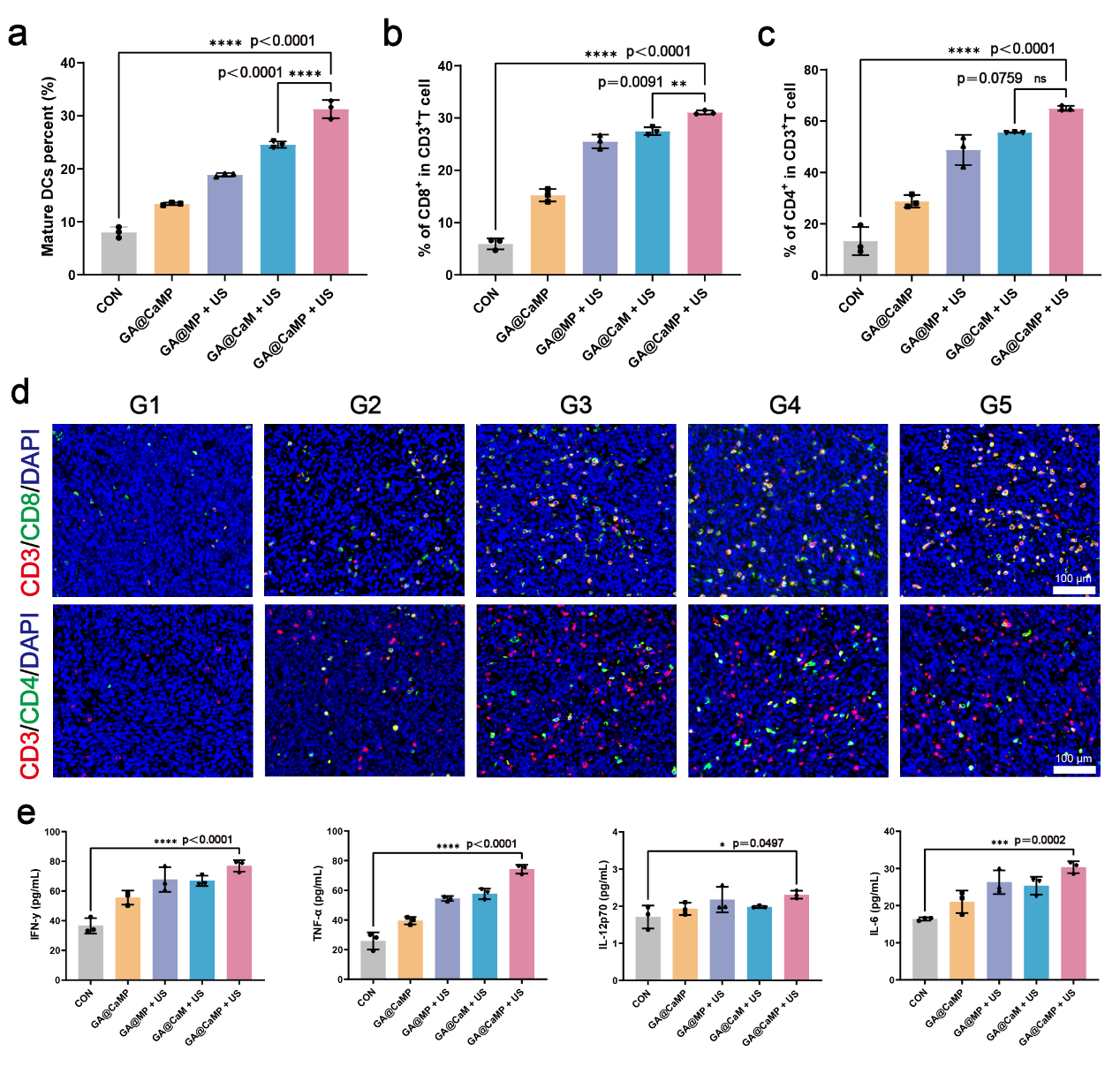


**Figure S24. In vivo analysis of immune effects.** **a)** Corresponding quantitative analysis of mature DCs in the recurrent tumors after various treatments, including Control, GA@CaMP only, GA@MP + US, GA@CaM + US, GA@CaMP + US (n = 3). **b)** Corresponding quantitative analysis of CD8^+^ and **c)** CD4^+^ cells in the recurrent tumors after various treatments, including Control, GA@CaMP only, GA@MP + US, GA@CaM + US, GA@CaMP + US (n = 3). **d)** Immunofluorescence images of proliferated CD8^+^ T cells and CD4^+^ T cells in recurrent 4T1 tumor tissue slices (G1: Control, G2: GA@CaMP only, G3: GA@MP + US, G4: GA@CaM + US, G5: GA@CaMP + US). **e)** Intra-tumor levels of IFN-γ, TNF-α, IL-12p70, and IL-6 (n = 3). Data are presented as Mean ± SD. Significance between multiple groups was calculated using one-way ANOVA and Tukey-Kramer multiple comparisons test. ****P < 0.0001, ***P < 0.001, **P < 0.01, *P < 0.05, ns: no significance.


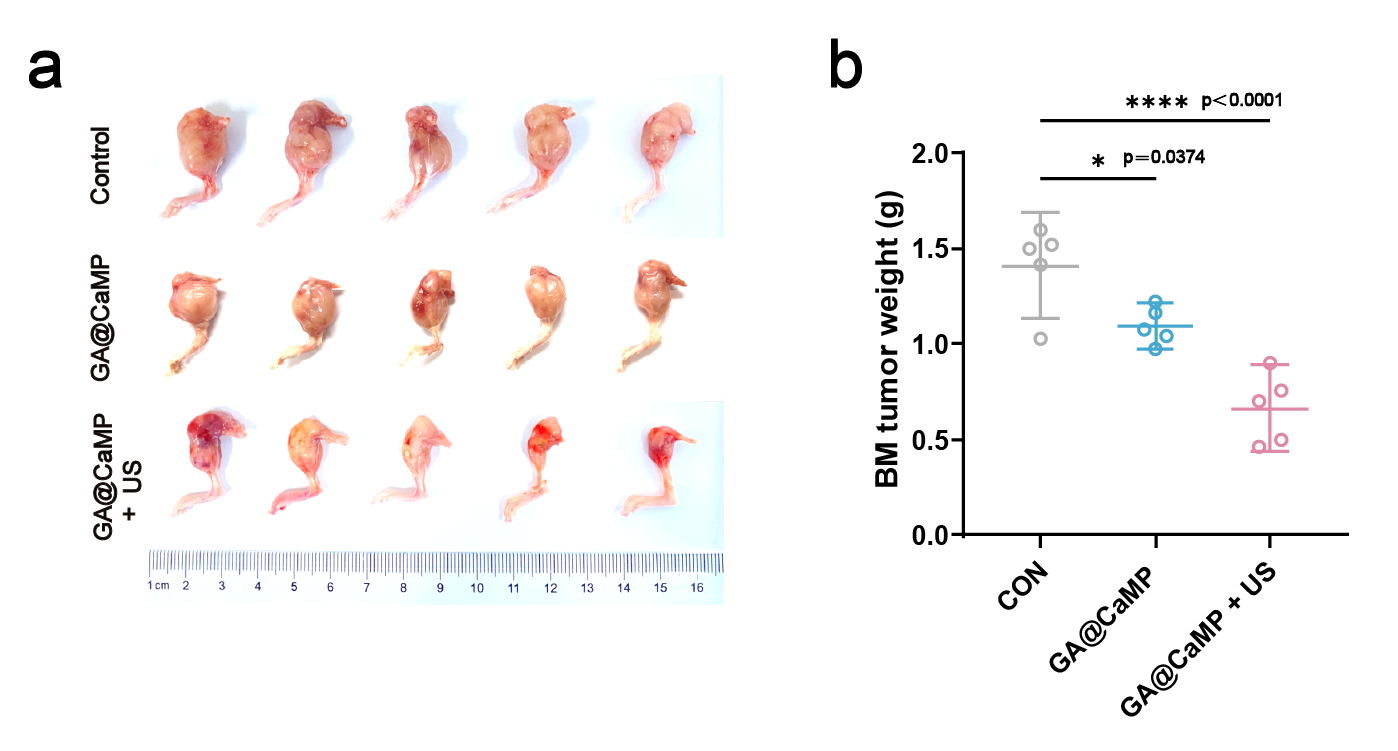


**Figure S25. a)** Photographs and **b)** average weight of the tumor-bearing tibiae collected after various treatments (Control, GA@CaMP, and GA@CaMP + US) in a tumor metastasis model for 14 days (n = 5). Data are presented as Mean ± SD. Significance between multiple groups was calculated using one-way ANOVA and Tukey-Kramer multiple comparisons test. ****P < 0.0001, ***P < 0.001, **P < 0.01, *P < 0.05, ns: no significance.


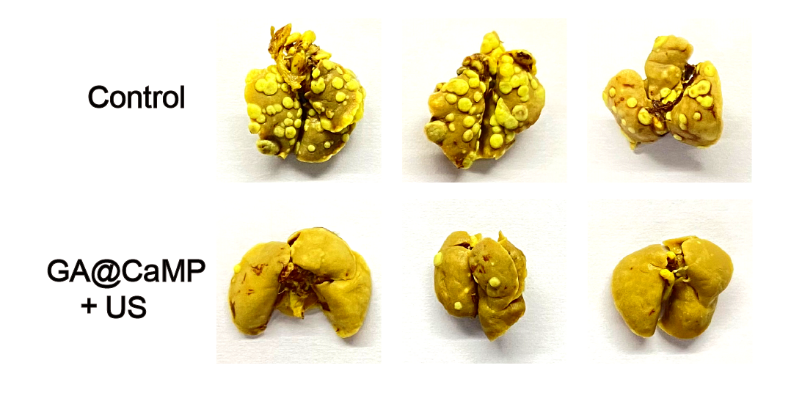


Figure S26. Pictures of lung metastatic nodules post various treatment methods (Control, GA@CaMP + US) (n = 3).


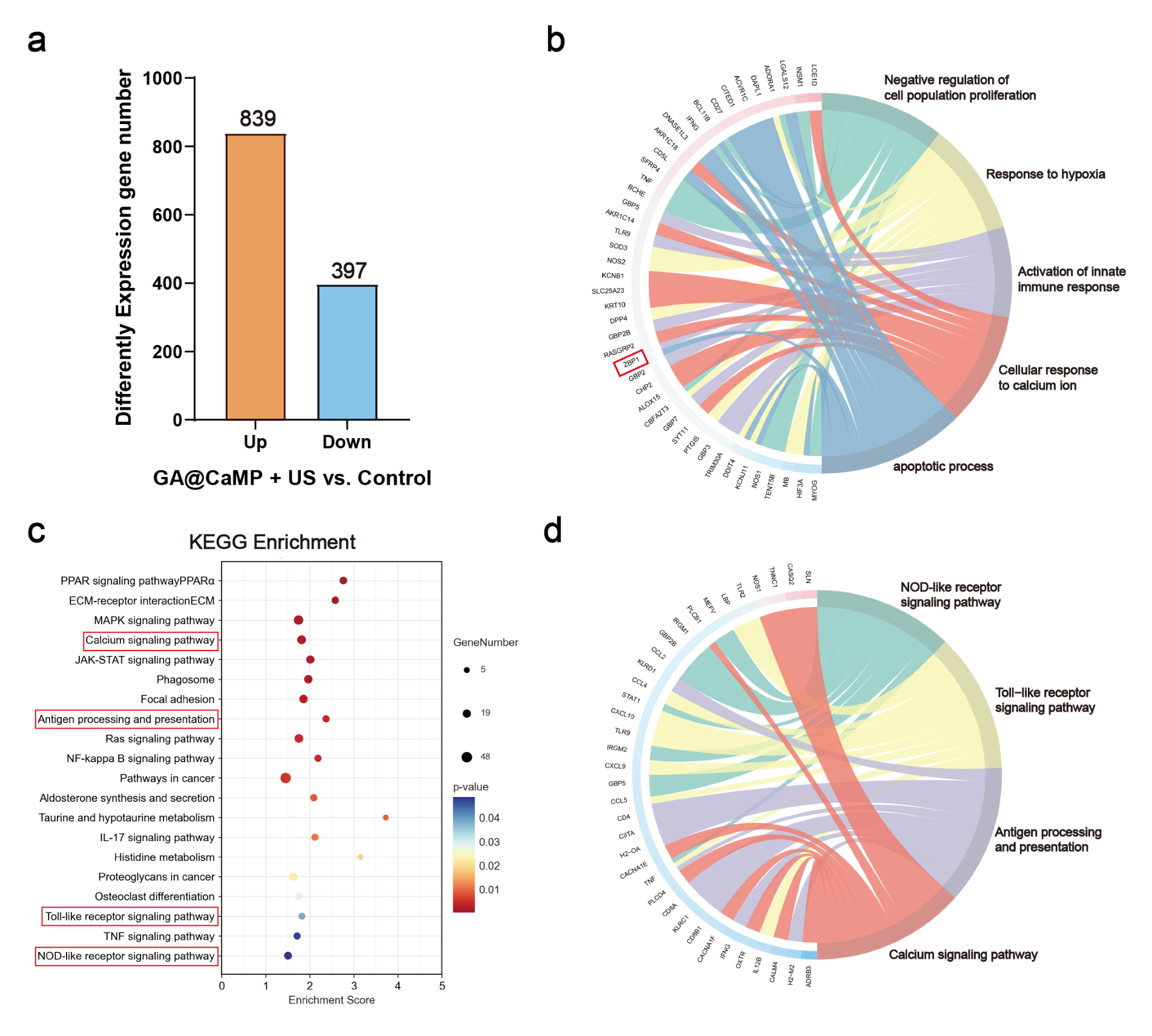


Figure S27. a) Number of gene alteration in the GA@CaMP + US group compared to the Control group. b) GO corresponding circle diagram for DEGs of interest after GA@CaMP + US treatments. c) KEGG enrichment analysis and d) corresponding circle diagram for DEGs of interest after GA@CaMP + US treatments.


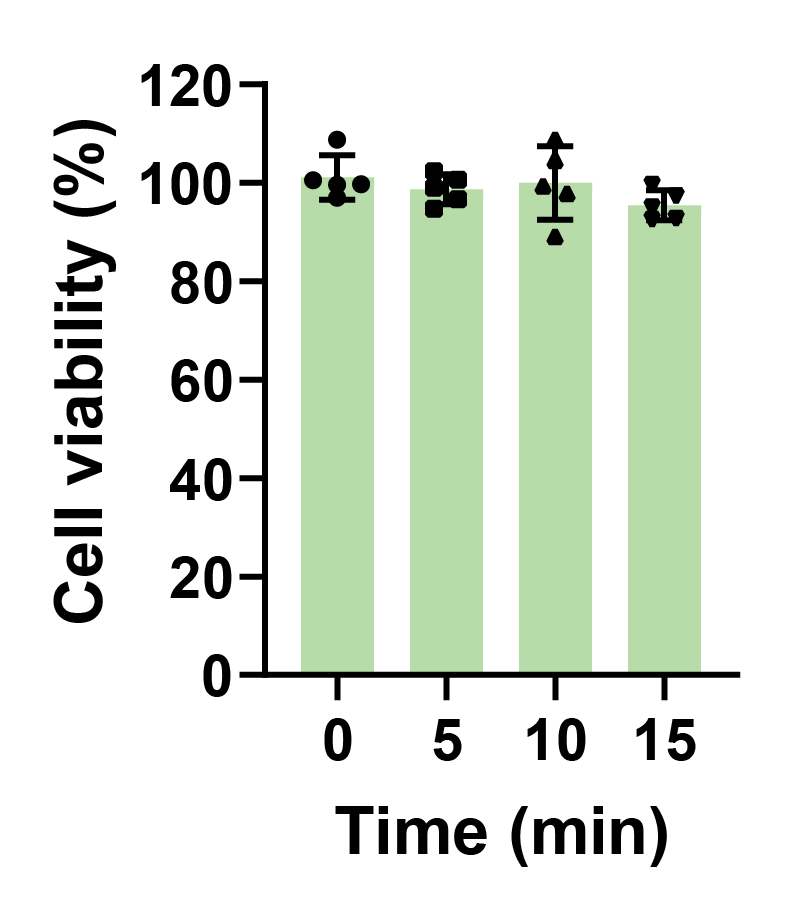


**Figure S28.** Cell viability of BMSCs seeded on GA@CaMP hydrogels after US exposure for different durations (0, 5, 10, and 15 minutes) (n = 5). Data are presented as Mean ± SD.


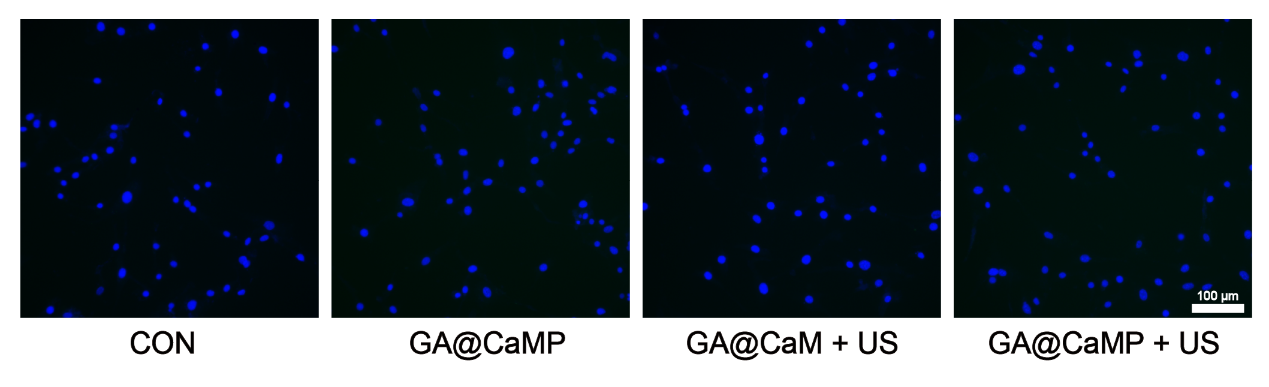


**Figure S29.** Measurements of ROS level using Fluorescence images of DCFH-DA staining after various treatments, including Control, GA@CaMP only, GA@CaM + US, GA@CaMP + US.


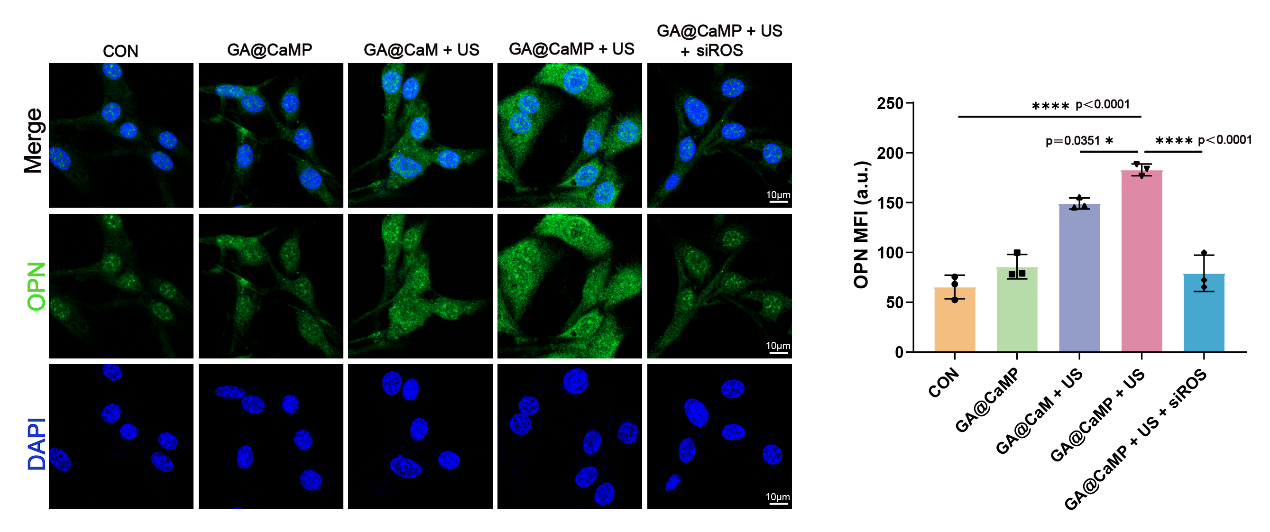


**Figure S30.** Immunofluorescence staining and quantitative analysis of OPN in BMSCs after various treatments, including Control, GA@CaMP only, GA@CaM + US, GA@CaMP + US, and GA@CaMP + US + siROS (n = 3). Data are presented as Mean ± SD. Significance between multiple groups was calculated using one-way ANOVA and Tukey-Kramer multiple comparisons test. ****P < 0.0001, ***P < 0.001, **P < 0.01, *P < 0.05, ns: no significance.


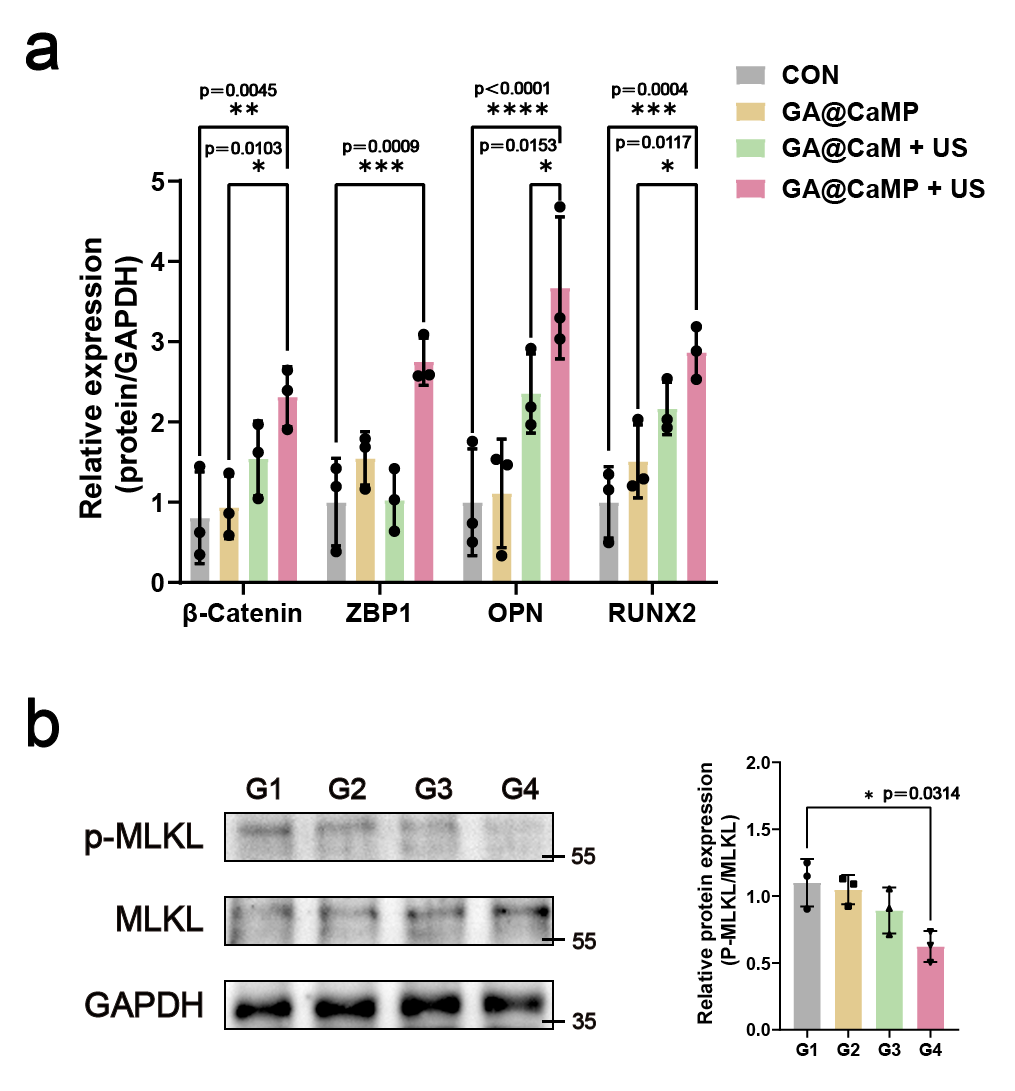


**Figure S31.** **a)** Related quantitative analysis of western blot analysis of osteogenesis-related proteins and ZBP1 protein (Control, GA@CaMP only, GA@CaM + US, GA@CaMP + US) (n = 3). **b)** Western blotting analysis and related quantitative analysis of necroptosis-related proteins expression levels in BMSC cells (G1: Control, G2: GA@CaMP only, G3: GA@CaM + US, G4: GA@CaMP + US) (n = 3). Data are presented as Mean ± SD. Significance between multiple groups was calculated using one-way ANOVA and Tukey-Kramer multiple comparisons test. ****P < 0.0001, ***P < 0.001, **P < 0.01, *P < 0.05, ns: no significance.


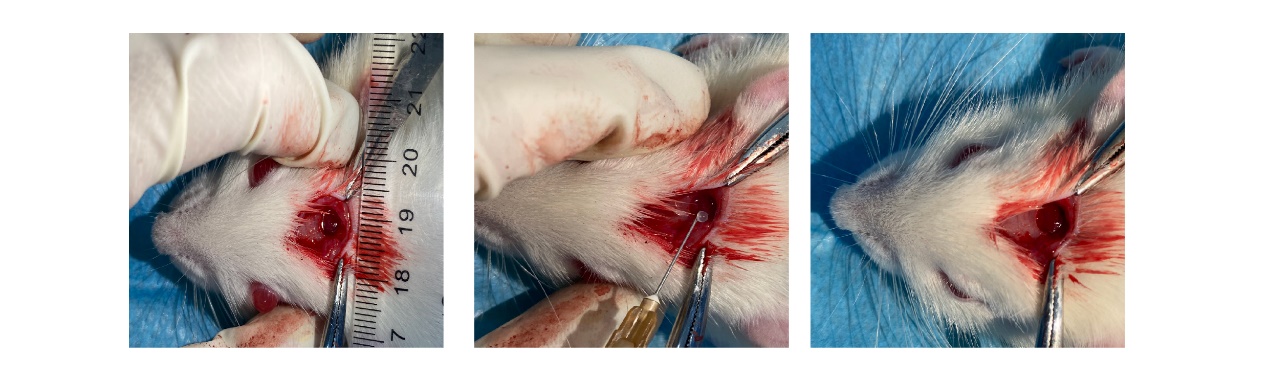


Figure S32. Photographs of hydrogels placed in a rat calvarial defect.


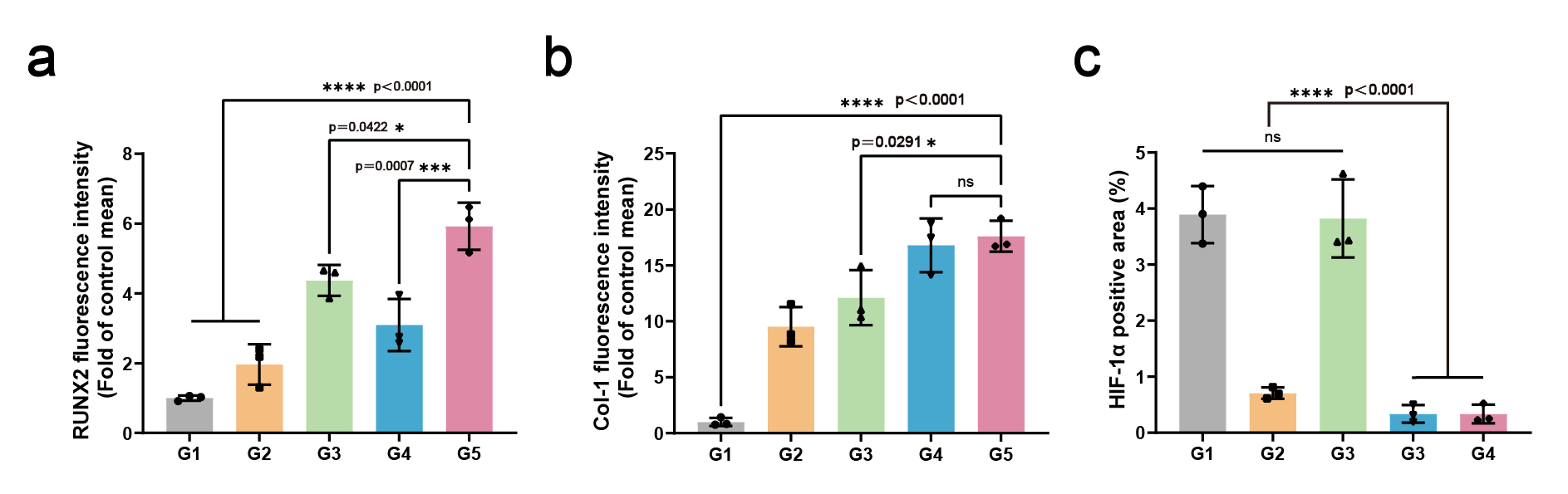


**Figure S33. a)** Quantitative analysis of RUNX2, and **b**) Col-1 immunofluorescence staining in the bone defect area. **c**) Quantitative analysis of HIF-1α-positive area in different groups (Control, GA@CaMP only, GA@MP + US, GA@CaM + US, GA@CaMP + US) (n = 3). Data are presented as Mean ± SD. Significance between multiple groups was calculated using one-way ANOVA and Tukey-Kramer multiple comparisons test. ****P < 0.0001, ***P < 0.001, **P < 0.01, *P < 0.05, ns: no significance.

Table S1. Preparation of GA hydrogels with varied monomer concentrations.

| Sample name | GelMA [mg] | AlgMA [mg] | Photoinitiator  [mg] | ddH_2_O [μL] |
| --- | --- | --- | --- | --- |
| 10 : 0 GA | 100 | 0 | 3 | 1000 |
| 7 : 3 GA | 70 | 30 | 3 | 1000 |
| 5 : 5 GA | 50 | 50 | 3 | 1000 |
| 3 : 7 GA | 30 | 70 | 3 | 1000 |
| 0 : 10 GA | 0 | 100 | 3 | 1000 |

Table S2. Primer sequences used in RT-qPCR analysis.

| Genes | Primers (F, forward; R, reverse) |
| --- | --- |
| Mouse-GAPDH | F: TCAACGGCACAGTCAAGG  R: TTAGTGGGGTCTCGCTCC |
| Mouse-RUNX2 | F: CATCCCAGTATGAGAGTAGGTGT  R: GCTCAGATAGGAGGGGTAAGAC |
| Mouse-OPN | F: TCTGAGGGACTAACTACGACCAT  R: TGGAAGAGTTTCTTGCTTAAAGTC |
| Mouse-Col-1 | F: CTGACTGGAAGAGCGGAGAG  R: CGGCTGAGTAGGGAACACAC |
